# Whole exome sequencing analysis of canine urothelial carcinomas without *BRAF* V595E mutation: Short in-frame deletions in *BRAF* and *MAP2K1* suggest alternative mechanisms for MAPK pathway disruption

**DOI:** 10.1371/journal.pgen.1010575

**Published:** 2023-04-20

**Authors:** Rachael Thomas, Claire A. Wiley, Emma L. Droste, James Robertson, Brant A. Inman, Matthew Breen

**Affiliations:** 1 Department of Molecular Biomedical Sciences, College of Veterinary Medicine, North Carolina State University, Raleigh, North Carolina, United States of America; 2 Comparative Medicine Institute, North Carolina State University, Raleigh, North Carolina, United States of America; 3 Office of Research (Biostatistics), College of Veterinary Medicine, North Carolina State University, Raleigh, North Carolina, United States of America; 4 Department of Surgery, School of Medicine, Duke University, Durham, North Carolina, United States of America; 5 Duke Cancer Institute, Duke University Medical Center, Durham, North Carolina, United States of America; 6 Center for Human Health and the Environment, North Carolina State University, Raleigh, North Carolina, United States of America; University of Bern Faculty of Veterinary Medicine: Universitat Bern Vetsuisse Fakultat, SWITZERLAND

## Abstract

Molecular profiling studies have shown that 85% of canine urothelial carcinomas (UC) harbor an activating *BRAF* V595E mutation, which is orthologous to the V600E variant found in several human cancer subtypes. In dogs, this mutation provides both a powerful diagnostic marker and a potential therapeutic target; however, due to their relative infrequency, the remaining 15% of cases remain understudied at the molecular level. We performed whole exome sequencing analysis of 28 canine urine sediments exhibiting the characteristic DNA copy number signatures of canine UC, in which the *BRAF* V595E mutation was undetected (UD^V595E^ specimens). Among these we identified 13 specimens (46%) harboring short in-frame deletions within either *BRAF* exon 12 (7/28 cases) or *MAP2K1* exons 2 or 3 (6/28 cases). Orthologous variants occur in several human cancer subtypes and confer structural changes to the protein product that are predictive of response to different classes of small molecule MAPK pathway inhibitors. DNA damage response and repair genes, and chromatin modifiers were also recurrently mutated in UD^V595E^ specimens, as were genes that are positive predictors of immunotherapy response in human cancers. Our findings suggest that short in-frame deletions within *BRAF* exon 12 and *MAP2K1* exons 2 and 3 in UD^V595E^ cases are alternative MAPK-pathway activating events that may have significant therapeutic implications for selecting first-line treatment for canine UC. We developed a simple, cost-effective capillary electrophoresis genotyping assay for detection of these deletions in parallel with the *BRAF* V595E mutation. The identification of these deletion events in dogs offers a compelling cross-species platform in which to study the relationship between somatic alteration, protein conformation, and therapeutic sensitivity.

## Author summary

Several human cancers, including melanoma and colorectal carcinoma, harbor a specific mutation within the *BRAF* gene, termed V600E. This mutation causes aberrant activation of the MAPK signaling pathway that normally regulates critical processes including cell growth, survival and proliferation. An equivalent mutation occurs in 85% of canine urothelial carcinomas (UC), the most common canine urogenital cancer. We performed DNA sequencing analysis of 28 canine UC cases that do not bear this mutation, to identify alternative mutations that may contribute to tumor development. We identified 13 specimens (46% of cases) harboring short in-frame deletions clustering elsewhere within the *BRAF* gene, or within *MAP2K1*, which also functions in the MAPK pathway. Importantly, equivalent deletions also occur in several human cancer subtypes, including certain leukemias and pancreatic carcinomas, and also Langerhans cell histiocytosis, resulting in structural disruption of the associated protein product. Critically, evidence is accumulating from human *in vitro* studies that tumors with *BRAF* or *MAP2K1* in-frame deletions are susceptible to different classes of small-molecule inhibitors, compared to those with *BRAF* V600E. Progress has been limited by the relative rarity of the human cancer subtypes in which these aberrations occur. With more than 60,000 dogs developing UC each year in the US, our findings reveal a strategy to overcome these limitations. By performing parallel functional studies of *BRAF* and *MAP2K1* in-frame deletions in canine UC we may greatly expand the volume of biological specimens and clinical data available for elucidating their significance for optimizing treatment protocols in both species.

## Introduction

Urothelial carcinoma (UC, also referred to as transitional cell carcinoma or TCC) is the most common canine urogenital cancer, with more than 60,000 dogs developing the disease each year in the US [1,2]. The incidence of the disease is markedly elevated in several popular breeds, most notably the Scottish Terrier (20-fold increased risk for invasive UC compared to mixed breed dogs) and also beagles, Shetland sheepdogs and West Highland white terriers (3 to 6-fold increased risk) [1,2]. Tumors typically are accompanied by non-specific clinical signs (including pollakiuria, hematuria, stranguria) that are shared with more common and/or readily treatable conditions such as urinary tract infection, bladder stones, benign polyps, and cystitis. As a consequence, canine UC is frequently diagnosed at an advanced stage. Ultimately the patient may lose the ability to urinate due to bladder outlet obstruction caused by the enlarging mass, requiring urgent decompression. At the time of diagnosis, the vast majority of tumors have already invaded the detrusor muscle, and median survival is typically less than 12 months with existing treatment options (reviewed in [1,2]).

Conventional cytological methods for diagnosis are often inconclusive due to limited sample availability, the variable appearance of normal epithelial cells, and the presence of neutrophilic infiltration in response to secondary bacterial infection. Ultrasonography has limited sensitivity for detecting small masses and urethral lesions, and for distinction between UC and benign lesions [3]. Histopathologic evaluation of tissue biopsies remains the gold standard, but is invasive, technically challenging, and expensive to perform. Our earlier molecular analyses of canine UC identified that the majority harbor a single base mutation in exon 15 of the canine *BRAF* gene [4,5]. This results in a valine-to-glutamic acid substitution within the activating domain of the BRAF serine/threonine protein kinase at codon 595, which is termed V595E, relative to transcript ENSCAFT00000006306 (also known as V588E, relative to ENSCAFT00000006305). Canine *BRAF* V595E is orthologous to the *BRAF* V600E variant found in several human cancer subtypes, which leads to constitutive activation of the RAS/RAF/MAPK pathway and concomitant upregulation of critical cellular processes including cell growth, survival and proliferation. To address the limitations of current diagnostic options, we developed an assay for detecting canine UC that uses droplet digital PCR (ddPCR) analysis to detect the *BRAF* V595E mutation in exfoliated tumor cells recovered from urine specimens [5]. Through analysis of hundreds of urine derived specimens we have shown that our *BRAF* V595E ddPCR assay detects canine UC with 85% sensitivity and >99% specificity. This approach has gained wide acceptance as a robust and reliable tool in both clinical and research settings [6–10], offering a powerful means for timely detection of UC, and identifying tumors that may be responsive to BRAF inhibitor therapy.

Given that ~85% of canine UC cases harbor the *BRAF* V595E mutation [4,5], published datasets are skewed heavily toward cases bearing this variant; consequently the biological and clinical significance of its absence in the remaining 15% of cases has not yet been established. This is in part due to the typically delayed diagnosis of UC in dogs, which confounds the ability to determine the precise order and relative clinical significance of individual molecular events. If they represent early-stage tumors in which the *BRAF* V595E mutation has not yet emerged, then their study has tremendous potential to yield methods for early detection and to identify somatic alterations that drive tumor progression. Conversely, if tumors without this mutation represent a distinct clinical subtype, their study may reveal other therapeutic targets and indicate a need for molecular subclassification for determining optimal treatment strategies.

Canine and human UC show many clinicopathologic parallels and there is evidence for shared genomic alterations that suggest conserved pathogenic mechanisms [11–14]. The oncogenic human *BRAF* V600E mutation is highly recurrent in several cancers, particularly malignant melanoma (~50% of cases) and also thyroid and colorectal carcinoma. Interestingly, however, while one in three human UC cases bear somatic alterations that activate the RTK/RAS/RAF pathway, only 3–4% exhibit variants within the *BRAF* gene, and only ~1% harbor the V600E mutation [15–18]. Additionally, over 75% of canine UC cases show evidence of muscular invasion at the time of diagnosis, compared to only 20–30% of human cases [2,19]. As a consequence, cross-species studies have largely involved comparison of canine muscle-invasive UC with more superficial human UC cases. One study of a very high-risk human UC cohort with a 2-year metastasis rate of 55% identified *BRAF* mutations in as many as 25% of cases [20]. The marked enrichment of mutated *BRAF* in these human cases provides an opportunity to examine whether canine UC cases bearing *BRAF* mutation more closely recapitulate the clinicopathologic and molecular characteristics of advanced/metastatic human tumors, and in turn, whether canine tumors with wild-type *BRAF* resemble lower-grade, localized disease in people. If so, then this would allow the more prevalent category of disease in each species to serve as a model for the rarer category in the other species, in a reciprocal manner.

To address these questions it is first necessary to generate a bank of genomic sequence data for canine UC cases that do not exhibit *BRAF* V595E mutation. In an earlier study we identified regional copy number gains of dog chromosome 13 (*Canis familiaris* chromosome 13, cfa13) and cfa36 in 97% and 84% of canine UC cases, respectively, while loss of cfa19 was evident in 77% of cases [11]. Over 75% of cases exhibited all three of these DNA copy number aberrations (CNAs) and >93% showed two or more. None of these alterations were evident in non-neoplastic controls, including cases of urinary tract infection, cystitis, and benign bladder polyps [21]. We developed a ddPCR assay for identification of these three CNAs and established that if two or more were detected in exfoliated cells recovered from canine urine specimens, the sensitivity and specificity to indicate the presence of a UC was >99% [5]. In this report we leverage the power of these molecular tools, in combination with whole exome sequencing (WES) analysis, to examine the genomic profiles of canine UC specimens without detectable *BRAF* V595E. Our primary goal was to determine whether these specimens exhibit somatic variants elsewhere within the *BRAF* gene, or in other components of the MAPK pathway, that define a likely pathogenic mechanism and a potential therapeutic target.

## Materials and methods

### Sample preparation and ddPCR screening of clinical specimens

Free-catch urine specimens were obtained from dogs exhibiting non-specific clinical signs including hematuria, stranguria, and pollakiuria. Owners of each dog provided consent for samples from their dog to be included in research studies, and associated clinical records were reviewed by a board-certified small animal veterinary internist (CAW). Exfoliated cells were pelleted by centrifugation, rinsed with sterile PBS, and processed for DNA extraction using a Maxwell RSC Instrument and Cell DNA Purification Kit (Promega, Madison WI). DNA samples were screened using ddPCR analysis for the presence of the dog *BRAF* V595E mutation, a T>A substitution at nucleotide 8,296,284 on cfa16 in the canFam3.1 reference genome assembly (denoted as cfa16:8,296,284) using the criteria described by [5]. Briefly, specimens in which *BRAF* V595E was detected (hereafter referred to as ‘POS^V595E^’) were analyzed to determine the percentage fractional abundance (FA) of the variant (calculated as [A/(A + B)] where A = number of copies/μl of the mutant allele and B = number of copies/μl of the wild-type allele). Specimens that exhibited fewer than five single-occupancy *BRAF* V595E mutant-positive droplets and a minimum of 5000 *BRAF* wild-type droplets were classified as ‘undetected’ (hereafter referred to as ‘UD^V595E^’). A detection threshold (DT) was determined for each sample (calculated as [5/(A + B]), providing a measure of the lowest *BRAF* V595E FA that the assay is capable of detecting for that individual sample. All specimens were then screened with the canine UC CNA ddPCR assay as described previously [21]. Briefly, this assay calculates the copy number of discrete regions of cfa13 and cfa36 relative to a region of cfa19. Specimens with a relative DNA copy number ratio >1.2 for both the cfa13/cfa19 signature and the cfa36/cfa19 signature were classified as CNA-positive (‘POS^CNA^’).

### Whole exome sequencing analysis

Two categories of POS^CNA^ samples were selected for further characterization using WES analysis. Twenty-eight UD^V595E^ specimens were selected in which the *BRAF* V595E mutant allele was undetected with a detection threshold < 0.1%. These were assigned the prefix ‘UD-‘. Eight POS^V595E^ specimens were selected that showed a high fractional abundance of the *BRAF* V595E mutation (>40%). These were assigned the prefix ‘POS-‘. For each specimen, ~25 ng of DNA were sheared to yield a mean fragment size of ~300 bp using a Covaris S220 Ultrasonicator (Covaris, Woburn MA). Libraries were prepared using a KAPA Biosystems HyperPrep Kit (Roche Nimblegen, Pleasanton CA) incorporating a unique dual-indexed barcode adaptor for each specimen. Due to the limited quantity of starting DNA available, size-selection was omitted. Solution-based target enrichment was performed independently (without pooling) for each sample using the Roche Nimblegen SeqCap EZ HyperCap workflow v.2.3 with Custom Developer Probes encompassing 52.9 Mb of dog exomic sequence (canine Exome-1.0 design, [22]). Library quality, fragment size range and yield were assessed before and after target enrichment using a 2200 TapeStation (Agilent Technologies, Santa Clara CA) and a Nanodrop One spectrophotometer (Thermo Scientific, Waltham MA), following the manufacturer’s recommendations. Blood-derived DNA samples from 12 non-neoplastic controls were processed in parallel using the same workflow. Also included was a specimen with a low fractional abundance of the *BRAF* V595E mutation (4.3%), to act solely as an internal control for comparison of ddPCR and WES data for this variant. This control sample was denoted ‘low-FA’.

Exome-enriched libraries were pooled at equimolar concentrations, loaded onto a NovaSeq 6000 S4 flow-cell (Illumina, San Diego CA) and sequenced with 150 bp paired-end reads (NC State University Genomic Sciences Laboratory, Raleigh NC). Fastq files were processed with a custom pipeline incorporating optimized tools from the Sentieon Genomics suite v.202010.02 (Sentieon Inc., San Jose CA) based on GATK best practices [23]. Briefly, raw reads were aligned to the canFam3.1 dog reference sequence assembly [24] using Sentieon bwa-mem, and duplicate reads were marked using Dedup and Locus Collector. Base quality score recalibration was performed using the dog dbSNP v151 database. Indel realignment was performed in parallel with variant calling using the haplotype-based Sentieon TNscope caller with default parameters [25]. This follows the general principles of the GATK Haplotype Caller and Mutect2 with enhanced sensitivity for detection of low-penetrance somatic variation [25,26]. TNscope analysis referenced a pool of all variants identified in the 12 non-neoplastic specimens, which were processed using the same library preparation workflow and sequenced on the same Illumina flowcell.

### Derivation of DNA copy number profiles from WES read depth data

DNA copy number was determined from WES read count data using the BAM MultiScale Reference (MSR) Algorithm within Nexus Copy Number (Biodiscovery, El Segundo CA). First, realigned bam files for the non-neoplastic control samples were pooled to create a common reference file using the Multiscale BAM Reference Builder module. Realigned bam files for each of the test DNA samples were then processed relative to the common reference file, using the MSR algorithm to generate pseudo-log ratios based on read depth.

Discrete regions of DNA copy number imbalance were defined using the FASST2 Segmentation algorithm within Nexus Copy Number, using default parameters. Briefly, the significance threshold for segmentation was set at 1x10^-6^, with a minimum of three probes per segment and a maximum spacing of 1Mb between adjacent probes before breaking a segment. The ratio thresholds for single copy gain and single copy loss were set at 0.18 and -0.18, respectively, and thresholds for high amplitude gains and losses were set at 0.6 and -1.0 respectively. Data analysis was restricted to autosomes due to the inclusion of dogs of both sexes in the study cohort.

### Variant filtering

Downstream variant filtering was performing using VarSeq v.2.2.3 (Golden Helix, Boseman MT). Briefly, variants that passed Sentieon TNscope’s variant calling filters were imported and annotated for gene content (Ensembl Genes release 100, [27]), sequence ontology [28,29] and overlap with previously identified canine germline and somatic variants (dbSNP build 151, [30,31]). The Cancer Gene Census database [32] was used to annotate the dog orthologs of genes within which mutations are causally implicated in human cancers. Variants that were evident in >5% of samples from the dataset generated by [31] were excluded from further analysis. Intergenic and intronic variants, and short tandem repeats were also excluded. Variant sites were required to show a read depth of 30 or greater, with a minimum of five reads for the alternate allele. Variants that met any of the following criteria were also excluded from further analysis, following GATK Best Practices recommendations [23] (for single nucleotide variants (SNVs): QD < 2, QUAL < 30, SOR > 3, FS > 60, MQRankSum < -12.5, ReadPosRankSum < -8 and for indels: QD < 2, QUAL < 30, FS > 200, ReadPosRankSum < -20). Statistical analyses were performed using JMP Pro v15.2.0 (SAS Institute Inc., Cary NC). After testing for normality, two-sample t-tests were performed to determine whether there was a significant difference in the mean number of mutations identified in POS^V595E^ and UD^V595E^ cases (p<0.05). The mean number of each category of mutations was compared between sample groups in the same manner. A two-sided F-test was used to determine whether the variance of these parameters within the two sample groups was significantly different (p<0.05). Selected variants were validated by conventional Sanger sequencing analysis of samples from the WES cohort.

### Validation and distribution assessment of selected variants

Four target regions were evaluated using capillary electrophoresis (CE) to detect sequence alterations based on differential amplicon size. Three of these regions harbored short deletion events in a subset of samples and are described in the Results section. PCR primer pairs were designed to flank each of these regions to permit discrimination between wild-type and mutant alleles based on differential amplicon size. Primers were designed such that there would be no overlap in the size of the resulting amplicons for the four target regions, when amplified from either tumor or normal (wild-type) DNA. The fourth target evaluated was the site of the *BRAF* V595E mutation. One primer was designed to the normal dog genome assembly reference sequence, with the 3’ end matching the wild-type T nucleotide. An additional 17 nucleotides of non-canine sequence were added to the 5’ end of this wild-type primer, to result in generation of a larger amplicon from the wild-type allele compared to the mutant allele. A second primer was designed with the 3’ end matching the A nucleotide of the V595E mutation, and with the fourth nucleotide from the 3’ end altered from the wild-type C into a G. This introduced a second site of mismatch with the wild-type allele, ensuring amplification only from the mutant allele. Both allele-specific *BRAF* V595 primers were tagged with the same 5’ fluorophore. A third, common primer was designed to the opposite DNA strand. This strategy was used to enable distinction between the wild-type allele and the mutant allele using a combination of allele-specific PCR and differential amplicon size, while using only a single fluorophore.

The forward primer for each of the four target regions was modified with a different 5’ fluorophore (6-FAM, VIC, NED or PET), and was purified by high-performance liquid chromatography (Thermo Fisher Scientific, Waltham MA). Target regions were amplified by conventional end-point PCR on non-neoplastic controls and test samples from the WES cohort, to verify the ability to detect known alterations. Each target region was amplified in independent reactions for each sample using Promega GoTaq Colorless Master Mix (final concentration 1x), 0.4 μM final concentration of each primer and ~5 ng template DNA, in a total volume of 10μl. Thermal cycling was performed using a C1000 Touch system (Bio-Rad, Hercules CA) using the following conditions: initial denaturation at 95°C for 2 min, followed by 30 cycles of 94°C for 30 s, 60°C for 30 s, 72°C for 30 s, and a final extension at 72°C for 30 min. Amplicons were pooled for each sample, and the resulting pool was diluted with an equal volume of ultrapure water. Next, 1μl of the diluted pool was combined with 10 μl HiDi formamide and 0.5 μl of GeneScan 600 LIZ Size Standard v.2 (Thermo Fisher Scientific), denatured and loaded onto an Applied Biosystems 3730 xl Genetic Analyzer. OSIRIS v2.16 software [33] was used to verify the expected size of the amplicon for each target region, by reference to the DNA size standard.

## Results

### Sample population

Urine-derived DNA samples from 36 dogs were analyzed using WES analysis, and their signalment data are provided in Table 1. Across all cases there was a bias toward female dogs (28 female, seven male, and one of unknown sex); however the proportion of dogs of each sex within sample groups (UD^V595E^ vs POS^V595E^) was not significantly different (p = 1.0, two-tailed Fisher’s exact test). Of the 36 dogs, 26 were reported by the owner as purebred, with 18 different breeds represented. Three breeds were represented by more than a single case (beagle, Boston terrier and Labrador retriever). The mean age of dogs in each sample group was not significantly different (10.6 years for UD^V595E^ cases and 11.6 years for POS^V595E^ cases, two-sample t test, p = 0.20).

**Table 1:** Signalment data and ddPCR assay values for the sample cohort. The table lists the owner-reported breed and sex of dogs included in the study (* indicates unknown sex), in addition to the age of the dog at the time of sample submission. The sample ID prefix UD denotes specimens in which the *BRAF* V595E variant was undetected, and the prefix POS denotes samples that tested positive for this variant by ddPCR analysis. For the 28 UD^V595E^ cases, the notation H/C after the sample name denotes that the diagnosis of UC was validated using conventional histopathology and/or cytology evaluation of tumor tissue (n = 7 cases). The notation US indicates the presence of a bladder mass on ultrasonography, with clinical signs and progression consistent with canine UC (n = 8 cases). Where detected, the VAF (from WES analysis) and FA (from ddPCR analysis) of the variant is shown as a percentage. The DT represents the lowest variant FA that was capable of being detected by the assay for each individual sample [5]. This value provides confidence that UD^V595E^ specimens are classified correctly down to a level of no greater than a single mutant allele among 1000 total alleles. The final two columns indicate the copy number ratio data derived using the UC CNA ddPCR assay. All 36 samples were classified as ‘positive’ for both DNA copy number signatures since they exceed the ratio threshold of 1.20 [21].

Sample code	Sex	Stated breed	Age (years)	*BRAF* V595E VAF (%)	*BRAF* V595E FA (%)	DT (%)	chr36/chr19 ratio	chr13/chr19 ratio
**UD-001 U/S**	FS	Border Terrier	11	UD	UD	0.04	2.95	1.64
**UD-003 H/C**	FS	Beagle	12	UD	UD	0.04	1.69	2.18
**UD-007 H/C**	FS	American Staffordshire Terrier	14	UD	UD	0.05	1.83	1.84
**UD-018**	FS	Labrador mix	12	UD	UD	0.04	1.80	1.80
**UD-027**	FS	Rat Terrier	14	UD	UD	0.03	2.05	1.54
**UD-033 U/S**	FS	Toy Poodle	12	UD	UD	0.04	1.51	2.11
**UD-049 U/S**	FS	Scottish Terrier	13	UD	UD	0.04	1.94	1.71
**UD-054**	FS	Beagle	8	UD	UD	0.04	1.71	1.95
**UD-081 H/C**	FS	Miniature Dachshund	9	UD	UD	0.08	2.31	1.68
**UD-082**	FS	Boston Terrier	13	UD	UD	0.04	2.26	1.71
**UD-084 U/S**	FS	Mix	10	UD	UD	0.05	1.70	3.10
**UD-085 H/C**	MN	Labrador Retriever	13	UD	UD	0.06	2.09	1.79
**UD-088 H/C**	MN	Fox Terrier	12	UD	UD	0.08	1.96	2.12
**UD-091 U/S**	FS	Great Dane	8	UD	UD	0.03	3.57	3.30
**UD-092 U/S**	MN	Terrier mix	11	UD	UD	0.03	3.60	2.52
**UD-097**	FS	Beagle	11	UD	UD	0.04	2.36	2.91
**UD-098**	FS	Boston Terrier	9	UD	UD	0.04	7.27	1.88
**UD-099 H/C**	FS	Beagle	8	UD	UD	0.04	3.15	1.88
**UD-100 H/C**	FS	Beagle/Terrier mix	13	UD	UD	0.05	2.12	2.07
**UD-102**	MN	German Shepherd mix	14	UD	UD	0.05	7.00	1.90
**UD-104**	FI	Bluetick Coonhound	13	UD	UD	0.05	3.23	3.46
**UD-105 U/S**	MN	Beagle	13	UD	UD	0.06	2.11	2.12
**UD-106**	FS	Golden Retriever/Collie mix	11	UD	UD	0.06	3.74	1.60
**UD-108**	MN	Bassett Hound	11	UD	UD	0.04	3.24	2.19
**UD-109 U/S**	FS	Beagle	12	UD	UD	0.05	2.62	2.38
**UD-110**	FS	Collie	12	UD	UD	0.06	3.96	3.03
**UD-112**	FS	Pomeranian	14	UD	UD	0.05	5.51	1.69
**UD-113**	FS	Beagle	13	UD	UD	0.06	3.58	2.45
**POS-124**	FS	Coton de Tulear	13	61.7	61.7	0.05	1.73	2.29
**POS-125**	FS	Labrador mix	9	65.1	60.2	0.11	2.90	1.82
**POS-127**	FS	Labrador/poodle mix	8	55.8	58.5	0.02	1.66	1.82
**POS-128**	FS	Miniature Schnauzer	10	55.3	55.4	0.02	2.41	1.63
**POS-131**	*	Labrador Retriever	12	49.5	53.0	0.03	2.52	1.63
**POS-134**	FS	Australian Cattle Dog	8	44.0	51.7	0.02	5.50	2.27
**POS-138**	FS	Mix	14	48.1	50.0	0.05	8.23	1.92
**POS-2027**	MN	Yorkshire Terrier/Shih Tzu mix	11	90.1	92.0	0.05	3.06	3.72

### Sequencing metrics and derivation of DNA copy number profiles from WES read depth data

On average 144 million read pairs were generated for each library with more than 90% of bases scored at Q30 or above. All samples had at least 100x coverage at >66% of bases across all intervals targeted by the 52.9 Mb whole exome capture bait panel (range 66.5–92.4%, median 87.9%). The mean coverage across all samples (urine-derived DNA samples and non-neoplastic controls) was 299x (range 157-433x, median 297x). DNA copy number profiles derived from WES read depth data were broadly comparable to those we reported previously in histologically verified canine UC using 26 kb resolution oligonucleotide-array comparative genomic hybridization (oaCGH) analysis [11,34]. S1A Fig shows an example of data derived from the same tumor DNA sample using both techniques. This demonstrates a high degree of conservation in the resulting profiles, ranging from whole chromosome aneuploidy through to complex segmental copy number alterations dispersed along the length of a chromosome.

S1B Fig shows penetrance plots comparing the genomic location and relative incidence of CNAs identified in two independent UC cohorts using oaCGH analysis [11] and WES analysis (this study). Despite the uneven probe distribution and limited genome coverage associated with WES data there was marked similarity in the DNA copy number profiles of POS^V595E^ and UD^V595E^ cases generated by both techniques, including the characteristic UC-associated signature of gain of cfa13 and 36, and loss of cfa19. These observations support the presence of exfoliated UC cells in the urine of the dogs included in this study.

### Overview of sequence variants identified from WES analysis

**Fig 1:**
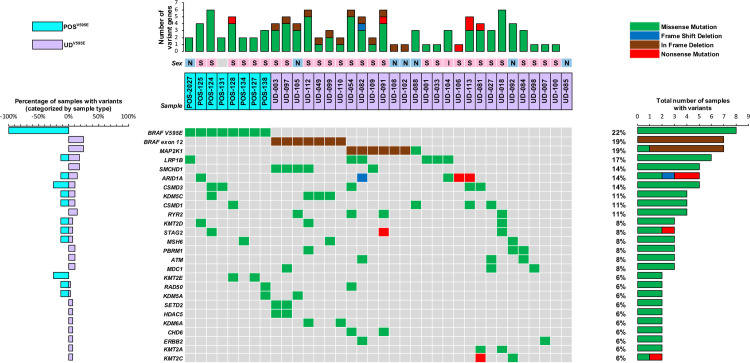
Overview of recurrently mutated genes within the study cohort. POS^V595E^ samples (n = 8) are denoted by aqua shading and UD^V595E^ samples (n = 28) by purple shading. The sex of each dog is indicated by a colored box above the sample code (females shown in pink, males in blue, and dogs of unknown sex shown in grey). Within that box, neuter status is shown as either N (neutered male), S (spayed female) or I (intact). Below this, 26 genes are listed that were mutated in two or more samples. The nature of the alteration in each sample is shown by a colored box (green = missense mutation, red = nonsense mutation, blue = frameshift deletion, brown = in-frame deletion). The plot to the right of the gene list indicates the overall percentage of all samples (n = 36) with alteration of each gene, and the nature of those variants. The plot to the left of the gene list compares the percentage of alterations among POS^V595E^ and UD^V595E^ samples. The horizontal plot at the top shows the number and nature of variants of these genes identified in each individual sample.

After filtering, the number of non-synonymous variants identified ranged from 80–295 per sample (mean = 164, median 157), equivalent to a mean tumor mutation burden of 3.1 mutations/Mb within exome capture regions. Within the eight POS^V595E^ samples the range was 80–242 variants per sample (mean = 153, median 161) compared to 109–295 in the 28 UD^V595E^ samples (mean = 167, median 156). These parameters were examined for normality by visual inspection to determine severity of skew and presence of outliers, and using the Shapiro-Wilk test we found no evidence of non-normality (p = 0.2298). There was no significant difference in the mean number of mutations detected in POS^V595E^ and UD^V595E^ cases (two-sample t test, p = 0.52), and there was no significant difference in the variability of the number of mutations observed between POS^V595E^ and UD^V595E^ cases (two-sided F test, p = 1.00). Similar comparisons for each category of mutation (frameshift, in-frame insertion/deletion, missense and gain/loss of stop) identified no significant differences in the mean or variability between POS^V595E^ and UD^V595E^ cases. Fig 1 provides a summary of non-synonymous mutations of key genes identified in two or more specimens within the sample cohort, which are described below. Additional details of variant frequency and distribution are provided in S2 and S3 Figs.

### Detection of somatic mutations within exon 15 of the dog *BRAF* gene

Mean sequence depth across all samples along the length of *BRAF* exon 15 ranged from 143x to 666x (median 373x). The V595E variant was identified in each of the eight POS^V595E^ samples, with variant allele frequency (VAF) ranging from 44.0% to 90.1%. The VAF for the low-FA control sample was 2.7% (total read depth 409x), compared to 4.3% from ddPCR analysis. The fractional abundance data for this variant derived from ddPCR and WES analyses showed a strong correlation in all eight POS^V595E^ samples and the low-FA control (R^2^ = 0.979, Table 1 and S4 Fig). There was no evidence of this variant in WES data from the remaining test samples and non-neoplastic controls, consistent with ddPCR analysis, and no other *BRAF* exon 15 variants were detected in any samples.

### Identification of *BRAF* sequence alterations outside exon 15

**Fig 2:**
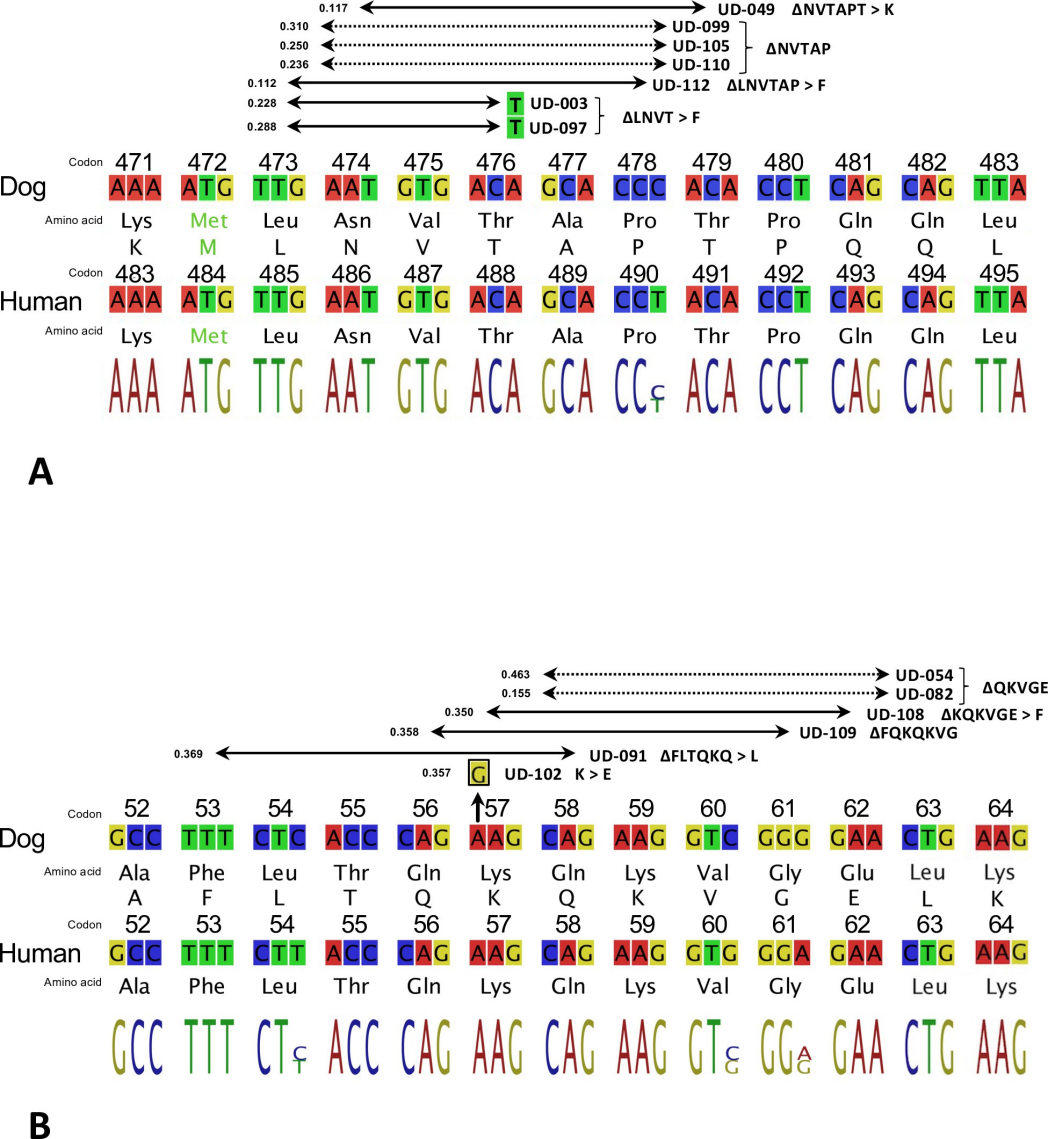
Summary of *BRAF* exon 12 and *MAP2K1* exon 2 deletions identified in UD^V595E^ specimens. a) Partial alignment of the deletion hotspot in canine *BRAF* exon 12 with its human ortholog shows complete conservation of amino acid sequence and only a single nucleotide difference. Horizontal arrows indicate the deleted regions identified within seven of the UD^V595E^ specimens, spanning either nine or 15 nucleotides (2/7 and 5/7 specimens, respectively). Deletions resulting in loss of entire codons are shown with dotted lines, and disruptive deletions with solid lines. Each region is annotated to indicate the amino acid sequence change resulting from the deletion (e.g. ∆NVTAP). The VAF is shown beside the left arrowhead for each case. Codon numbering in the dog *BRAF* gene is assigned relative to Ensembl Transcript ENSCAFT00000006305. b) The canine and human deletion hotspots in *MAP2K1* exon 2 also show complete conservation of amino acid sequence, with three nucleotide differences. Five samples showed deletions spanning 15 nucleotides. A sixth sample (UD-102) showed a single base change (A > G) resulting in a K57E alteration. Codon numbering in the dog *MAP2K1* gene is assigned relative to Ensembl Transcript ENSCAFT00000043934.

Short deletions within *BRAF* exon 12 were identified in 7/28 (25%) UD^V595E^ cases, clustered within a 17 bp interval spanning codons 473–479 (relative to Ensembl transcript ENSCAFT00000006305, cfa16: 8,276,702–719; Figs 2 and S5). Each was an in-frame deletion of either nine or 15 nucleotides (2/7 and 5/7 cases, respectively). Three samples (UD-099, UD-105 and UD-110) shared the same deletion of 15 nucleotides, which is hereafter denoted ∆NVTAP based on the single letter codes for the five deleted amino acids. The remaining four deletions were disruptive, resulting in ∆LNVT > F (UD-003 and UD-097), ∆LNVTAP > F (UD-112) and ∆NVTAPT > K (UD-049).No deletions were identified within this region in any of the remaining UD^V595E^ samples, nor in any POS ^V595^ cases or control samples. No other variants were evident elsewhere within the exons of the canine *BRAF* gene in any UC case or control specimen.

### Sequence evaluation of upstream genes from the RAS/RAF/MAPK pathway

Other members of the RAF family were assessed for evidence of somatic alteration. A single non-synonymous SNV was located in *ARAF*, a missense C>T substitution within the kinase domain in exon 16, resulting in P528S (POS-124). No putative somatic variants were detected in *RAF1/CRAF* or in members of the RAS gene family (*HRAS*, *KRAS*, *NRAS*). Growth factor receptors and their ligands were then examined. The *EGF* gene was disrupted in a single sample, a premature stop codon in exon 14 (W704*, POS-128). Sample POS-124 showed two missense substitutions within exon 28 of the associated receptor gene *EGFR* (H1069Y and P1088A). Two missense variants were identified in the receptor tyrosine kinase gene *ERBB2*, one each in samples UD-007 (exon 2, L37P) and UD-082 (exon 7, G292R). Single missense mutations were identified in each of *FGFR1* (UD-081), *FGF5* (UD-104) and *FGF6* (UD-088). No variants were identified in genes encoding *PDGF*/*PDGFR* or *VEGF*/*VEGFR*.

### Assessment of downstream MAPK pathway genes

Analysis of downstream MAPK pathway members identified six samples exhibiting short in-frame deletion events in *MAP2K1* (which encodes the MEK1 protein kinase). These were restricted to UD^V595E^ cases that showed no deletions in *BRAF* exon 12 (6/28, 21%). Five were 15 bp deletions in *MAP2K1* exon 2, and were clustered within the interval cfa30:30,720,179–206 spanning codons 53–62 (relative to Ensembl Transcript ENSCAFT00000043934; Figs 2 and S5). Of these, one was a disruptive in-frame deletion (ΔFLTQKQ > L, UD-091) and the remainder were non-disruptive, comprising ΔQKQKVG (UD-109), ΔQKVGE (UD-054 and UD-082) and ΔKQKVGE (UD-108).

Also within this interval was a single missense substitution resulting in K57E (UD-088). The sixth *MAP2K1* deletion event was elimination of 6 bp within exon 3 leading to ΔPA (codons 105–106, UD-102). No other alterations were identified in *MAP2K1*, and none were evident in *MAP2K2* (MEK2) or the downstream pathway members *MAPK1* (ERK) or *MAPK3* (ERK1). RAS can also regulate critical cellular processes via the P13K/AKT/mTOR signaling cascade and so key members of this pathway were examined. A single instance of *TP53* mutation was identified, within exon 7 (UD-091) resulting in C230S. No variants were evident in the key cancer genes *PTEN*, *MTOR*, *AKT1*, *TSC1/2* or *MDM2*, or in genes encoding components of PI3K.

### Mutations of epigenetic modifiers, DNA repair genes and chromatin organizers

Epigenetic modifiers showed evidence of recurrent alteration. Among histone-modifying genes, missense mutations were identified in the lysine demethylase genes *KDM1A* (POS-128, three variants), *KDM5A* (POS-138, UD-105), *KDM5C* (POS-124, UD-049, UD-099 and UD-112) and *KDM6A* (UD-110 and UD-112). Missense mutations were also identified in the lysine methyltransferase genes *KMT2A* (UD-018) and *KMT2C* (UD-092), while sample UD-081 showed a missense mutation in the former and a premature stop in the latter. *KMT2D* showed missense mutations in UD-018 (two variants), UD-112 and POS-125, and *KMT2E* was mutated in POS-127 and POS-128. Two samples (UD-003, UD-097) shared the same missense mutation in *SETD2* (also known as *KMT3A*), resulting in S509C, and *EZH2* (also known as *KMT6A*) was mutated in UD-091. Missense mutations also occurred in the histone acetylation genes *EP300* (UD-113), *HDAC5* (UD-003 and UD-097, which shared the same alteration), *HDAC7* (UD-100) and *HDAC9* (UD-018), and in the histone acetyltransferase gene *KAT6B* (UD-018). Key DNA methylation genes, including *DNMT3A* and *TET2*, showed no evidence of mutation.

Among DNA repair genes three showed alterations in more than a single sample. The mismatch repair gene *MSH6* showed missense mutations in three samples, within exon 4 (UD-092, two variants, and UD-099) and exon 5 (POS-134). The double-strand break repair gene *RAD50* was mutated in both UD-054 and POS-138. Also of note, missense mutations of the *ATM* serine/threonine kinase gene, an initiator of DNA damage response, occurred in three cases (UD-027, UD-082 and UD-084). *MDC1* (Mediator of DNA Damage Checkpoint 1) also showed missense mutations in three UD^V595E^ samples (UD-027, UD-097 and UD-098).

There was also recurrent disruption of chromatin remodeling genes. Loss of function alterations of *ARID1A* were identified in three samples, comprising nonsense mutations in exon 1 (UD-106) and exon 4 (UD-113), and a frameshift deletion in UD-082 (exon 20). Missense mutations of *ARID1A* were detected in a further two samples, within exon 3 (POS-125) and exon 12 (UD-104). A nonsense mutation was identified in *SMARCC1* (UD-113), while three samples had missense mutations in SWI/SNF complex gene *PBRM1* (UD-084, UD-092 and UD-112). Members of the Chromodomain Helicase DNA Binding Protein family were recurrently altered, including *CHD4* (UD-092), *CHD5* (POS-125), *CHD6* (UD-091 with two mutations, and UD-054) and *CHD7* (UD-084). Four UD^V595E^ samples (UD-003, UD-097, UD-105 and UD-109) shared the same mismatch alteration in exon 2 of *SMCHD1* (Structural Maintenance Of Chromosomes Flexible Hinge Domain Containing 1) resulting in N94T, and a fifth was identified in UD-112 (exon 7).

### Additional genes exhibiting recurrent alterations

Aside from members of the Ras/Raf/MAPK pathway, *LRP1B* (LDL Receptor Related Protein 1B) was the most frequently disrupted gene identified in the present study. Six missense mutations were identified among five samples within this gene (two variants in POS-2027 and one each in UD-001, UD-033, UD-054, UD-082 and UD-104). *CSMD3* (CUB And Sushi Multiple Domains 3) was also among the most frequently mutated genes within the present study, with missense mutations evident in five samples (POS-124, POS-131, UD-054, UD-081 and UD-113 (two variants). The related gene *CSMD1* showed missense mutations in four additional samples (POS-128, UD-027, UD-088 and UD-113). Four UD^V595E^ samples (UD-018, UD-054, UD-091 and UD-105 with two variants) exhibited missense mutations within the *RYR2* gene (Ryanodine Receptor 2) gene. *STAG2* (Stromal Antigen 2) was altered in three samples (missense mutations in UD-018 and POS-124, and a gain of stop in UD-091). Notably, among these four recurrently mutated genes, no samples shared the same variant, nor was the same codon altered in multiple samples.

### Development of combinatorial assays for detecting in-frame deletions in *BRAF* and *MAP2K1*

**Fig 3:**
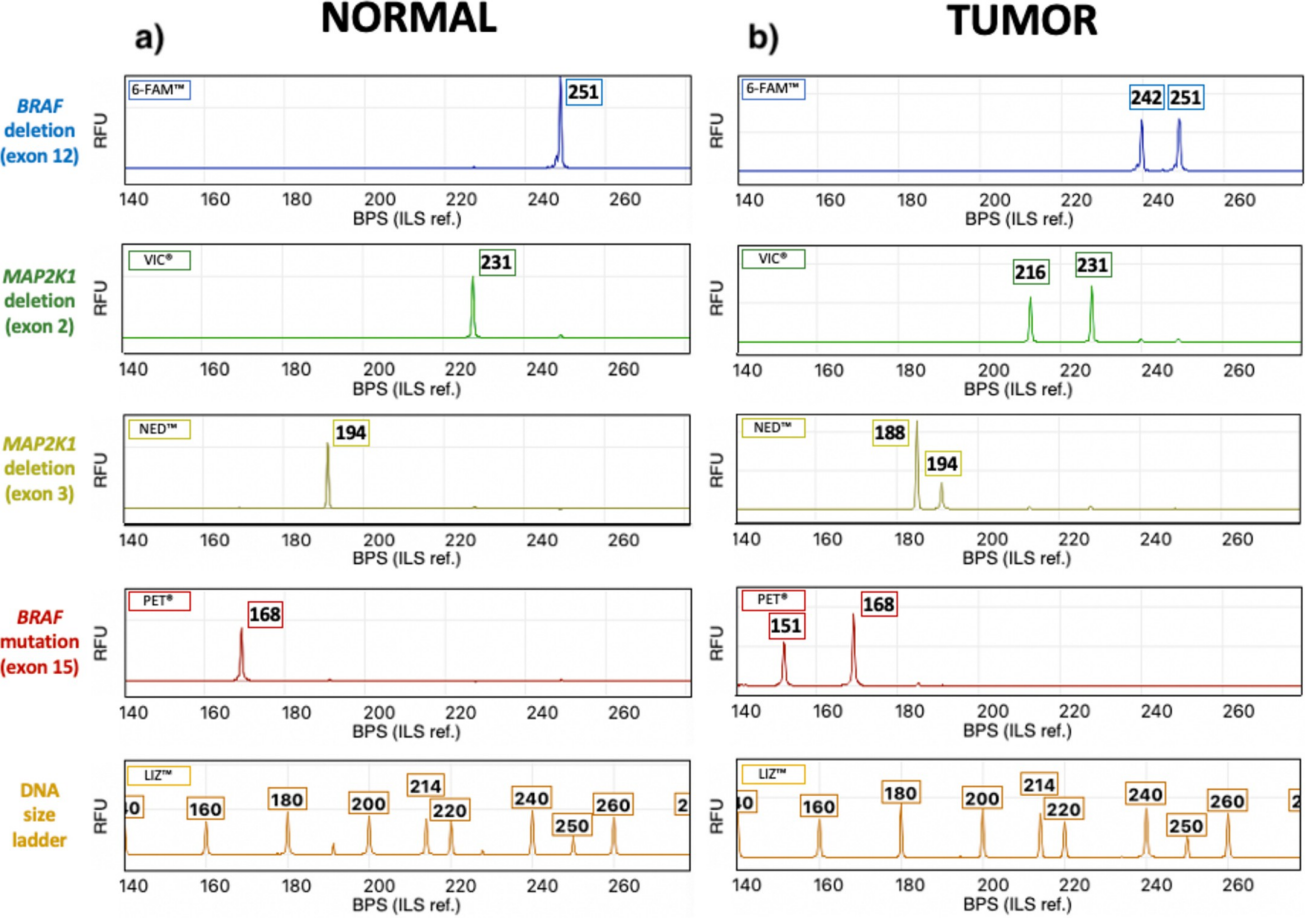
Detection of DNA sequence variants within the *BRAF* and *MAP2K1* genes using capillary electrophoresis. a) Fluorescent peaks represent amplicons generated for each of the four genomic targets in a normal (non-neoplastic) control sample. Numbers above each peak indicate the size of the amplicon in basepairs, determined by reference to the DNA size ladder shown at the bottom. In the normal control sample, only one peak is evident for each of the four targets, consistent with a normal wild-type sequence. b) Corresponding results obtained from tumor samples. The upper three plots show a second peak, indicating the presence of a smaller amplicon resulting from a deletion event. In the fourth plot, the second peak indicates the presence of a mutant *BRAF* V595E allele. Note that each of the four plots on the right side of the figure is derived from a different tumor specimen, since studies to date have shown these sequence alterations to be mutually exclusive events. BPS = base pair size RFU = relative florescence units.

Regions of *BRAF* exon 12 and *MAP2K1* exons 2 and 3 were investigated using novel CE assays to verify the presence of the deletions identified in sequenced samples, and to determine their frequency in additional specimens. A fourth assay was developed for distinction between wild-type and mutant alleles at the *BRAF* V595E locus. Primer sequences and observed amplicon sizes are provided in S1 Table. Fig 3A shows an example of the resulting data obtained using DNA isolated from a normal (non-neoplastic) specimen. A single peak of fluorescent signal was present for each amplified product, the size of which was consistent with wild-type sequence. Fig 3B shows data from specimens that showed sequence alterations within these intervals in WES analysis. CE assay results were consistent with the presence of the *BRAF* V595E mutation in POS-138, a 9 bp deletion within *BRAF* exon 12 in UD-097, a 15 bp deletion within *MAP2K1* exon 2 in UD-109 and a 6 bp deletion within *MAP2K1* exon 3 in UD-102. The remaining 10 samples that showed *BRAF* exon 12 or *MAP2K1* exon 2/3 deletions in WES analysis were analyzed in the same manner, and the resulting data from both approaches were fully concordant.

## Discussion

### Aims of the study

Previous studies have identified an activating *BRAF* V595E mutation in 85% of canine UC cases [4,5], highlighting the MAPK pathway as a compelling target for inhibitor therapy. The orthologous human V600E mutation causes the BRAF protein to mimic the conformational changes that normally occur during dimerization, allowing it to act as a monomer without prior need for RAS activation. This typically results in a ~500-fold increase in kinase activity compared to wild-type BRAF (reviewed in [35,36]). V600E can be targeted by BRAF monomer inhibitors such as vemurafenib and dabrafenib that act on the so-called ‘αC-out/DFG-in’ structural conformation of the active protein kinase. Ongoing studies are investigating whether vemurafenib induces a similar therapeutic effect in canine UC cases bearing the *BRAF* V595E variant [9]. The remarkably high incidence of *BRAF* V595E mutation in canine UC has resulted in a biased focus on genomic profiling of tumors bearing this variant. This study aimed to identify recurrent variants among cases without the *BRAF* V595E variant that have potential to extend existing diagnostic strategies and/or to provide alternative candidates for additional trials of targeted therapies.

### Comparison of methods for detection of *BRAF* V595E mutations

ddPCR and WES data showed complete correlation in terms of *BRAF* V595E variant status, with each sample classified as either positive or undetected by both methods. Quantitative assessment of the VAF for this site in WES data showed strong correlation with the FA determined by ddPCR analysis across a wide range of values, supporting the validity of integrating results from both methods for this variant. No alternative mutations of this site were identified that might result in a similar phenotype but fail to be detected using the ddPCR assay. Furthermore, since no other *BRAF* exon 15 variants were identified in any samples there is no evidence that absence of a positive ddPCR result in UD^V595E^ cases reflects failure to amplify the mutant allele due to sequence mismatches with either primers or probe.

BRAF is one of three evolutionarily conserved serine/threonine kinases that link RAS to MEK. The remaining two paralogs, ARAF and RAF1 (also known as CRAF) are less potent activators of MEK, and are rarely mutated in human cancers [37]. Furthermore, while *BRAF* mutations are reported in only 1.4% of human urinary tract cancers, the V600E variant constitutes 95.9% of amino acid substitutions found within this gene across all human cancers [38]. The absence of *BRAF* point mutations other than V595E in this study, and the lack of evidence for recurrent alterations in *ARAF* and *RAF1*, is globally consistent with observations in human cancers. We therefore investigated other MAPK pathway-related genes for mutations that might also confer susceptibility to inhibitor therapies.

### Evaluation of upstream genes within the Ras/Raf/MAPK pathway

RAS acts as the intermediary between the extracellular stimulus and the RAF kinase, and is encoded by three isoforms (*HRAS*, *KRAS* and *NRAS*). These are among the most frequently mutated genes across all human cancers [38]. In a previous study, Sanger sequencing analysis of 29 POS^V595E^ and 10 UD^V595E^ canine UC samples failed to identify any mutations of these genes [39]. While the use of this methodology would have limited the ability to detect low frequency variants, the absence of *HRAS*, *KRAS* or *NRAS* mutations in the present WES study supports these earlier findings.

Human invasive UC often shows aberrations of receptor tyrosine kinases and ligands that act as the initial stimulus for MAPK pathway activity, offering several potential therapeutic targets. Activating alterations involving the ErbB receptor tyrosine kinase family are recurrent, primarily as a result of somatic mutation of *ERBB2* and *ERBB3* (~10% of cases) and also increased copy number of *ERBB2* and *EGFR* [18,38]. Mutations of *EGF* and *EGFR* were each found in only a single sample from our cohort and did not coincide with sites that are altered frequently in human cancers [38]. No variants were identified in ErbB family members. Prior studies have, however, reported frequent upregulation of EGFR and ERBB2 in canine UC [13,40–44]. Immunohistochemistry studies have detected overexpression of the Her2 protein produced by *ERBB2* in ~60% of cases [42,45]. More recently, increased DNA copy number of *ERBB2* has been reported in 35% of dog UC cases [46], which suggests that upregulation may be driven by aberrant gene dosage rather than somatic mutation. *FGFR3* is also among the most frequently mutated genes in human UC, with somatic variants identified in ~26% of all cases [38]. Notably, several studies have shown a significantly lower incidence of *FGFR3* mutation in human high-grade invasive UD compared to low-grade cases (8–12% and 79–83% of cases, respectively) [18,47,48]. The absence of *FGFR3* mutation in the present study concurs with the fact that the vast majority of canine UC cases are invasive at the time of diagnosis.

### Evidence for disruption of related pathways

Altered receptor tyrosine kinases such as EGFR and ERBB2 can also stimulate oncogenic activation of the PI3K/AKT/mTOR pathway, which interplays with the Ras/Raf/MAPK cascade in regulation of critical cellular processes. Disruption of the RTK/Ras/PI3K pathway occurs in ~72% of human high grade invasive UC [18]; however in many cases this is due to aberrant PI3K [18,38,49]. *PIK3CA* is the most commonly mutated of the PI3K subunit genes across all human cancers, and while infrequent in UC (5–10% of all UC cases and 20% of high-grade invasive cases), mutations of *PIK3CA* are associated with a poor prognosis [18,38,49]. The vast majority of *PIK3CA* somatic alterations occur within two hotspots located in exon 9 (codons E542K and E545H) and one in exon 20 (H1047R/L). These regions are highly conserved between the human and canine genomes, and are often mutated in canine hemangiosarcomas and mammary carcinomas [50,51]; however *PIK3CA* was not mutated in our canine UC cohort, nor were genes encoding other components of PI3K.

Interestingly, the pattern of *TP53* alteration in human UC shows the inverse of *FGFR3*, with *TP53* mutations reported in ~50% of high-grade invasive tumors compared to 15% of low-grade non-invasive tumors [18,47,48,52]. Overall, it is estimated that the p53/cell cycle pathway is inactivated in approximately 90% of human UC, primarily through mutation of *TP53* and/or *RB1* [18,53]. In our study, no instances of *RB1* mutation were identified, and only a single *TP53* mutation was evident. This is consistent with a recent study of 11 cases of canine UC in which no *TP53* mutations were identified using WES and RNAseq analysis [54].

### Further comparisons with prior studies of canine and human UC

Despite the use of independent sample cohorts and different analytical strategies, our study showed several parallels with prior reports of the genomics of canine UC. As with our specific comparison of ddPCR and WES analysis for detecting the *BRAF* V595E variant, this replication provides confidence for the integration of data from different sources. This is of particular importance where sample resources are restricted due to the relative infrequency of the diagnosis, and where the frequency of any given variant within the population is low. Several earlier studies of canine UC have reported a low incidence of recurrently mutated genes other than *BRAF*. In one recent study of 11 canine UC samples, mutations of protein-coding regions were identified in 32 cancer-related genes; however only five of these (*BRAF*, *LRP1B*, *CUL3*, *RNF213* and *MSH2*) genes were mutated in more than a single sample, and only two were mutated in more than two samples (*BRAF* 4/11 samples, *LRP1B* 3/11 samples) [54]. Similarly, prior canine UC reports noted absence of variants in genes that are frequently altered in human UC, including *CDKN2A*, *FGFR3*, *HRAS*, *MDM2*, *PIK3CA* and *TP53* [54], which was consistent with our findings in the present study.

### *CSMD3* –a candidate for association with UC pathogenesis

Within the present study, the detection of variants within genes that were recurrently mutated in prior canine UC studies further elevates their candidacy for involvement in disease pathogenesis, particularly where there is similar evidence from human tumors. Among these is *CSMD3* (CUB and Sushi multiple domains 3), which encodes a protein whose function is not yet understood. *CSMD3* was mutated in five samples from the present study (14% of cases, comprising 2/8 POS^V595E^ and 3/28 UD^V595E^ samples), and was also highlighted as a recurrently mutated gene in bladder cancers of both dogs and people [14]. While rarely altered in human cancers in general (<0.5% of all cases), the incidence of *CSMD3* disruption in UC (~14% of cases) is among the highest across cancer subtypes, along with ovarian, breast, gastric and colon carcinomas [17,38]. A recent study in human ovarian cancers showed a significant correlation between *CSMD3* mutation, elevated tumor mutation burden and shorter overall survival. Similar observations have been reported for *CSMD1* in human gastric cancer [55], which was also mutated in multiple samples in the present study (11% of cases, 1/8 POS^V595E^ and 3/28 UD^V595E^ samples). The relatively high incidence of *CSMD3* mutation in both human and canine UC, and the correlation with outcome in other human cancers, renders this gene a worthy candidate for an association with UC pathogenesis.

### *LRP1B* and *RYR2* variants may yield positive predictors of immunotherapy response

Four UD^V595E^ samples (14%) exhibited missense mutations within the *RYR2* gene, a calcium channel component. Ramsey identified *RYR2* mutations via RNAseq analysis of seven canine UC cases, including one that was orthologous to a human UC driver mutation candidate [14]. Recurrent mutations of *RYR2* have been reported in several human carcinomas, including squamous cell carcinoma of the oral cavity [56] and lung [57], esophageal adenocarcinoma [58] and breast cancer [59]. Collectively, these studies present evidence that *RYR2* mutation predicts a positive response to immunotherapy in multiple human cancers. Canine invasive UC has been identified as a pertinent model for driving the development and evaluation of novel immunotherapeutic agents, based on its cellular and genomic composition [1]. The *RYR2* gene should therefore be evaluated for potential predictive value in this context.

Sporadic missense mutations of *LRP1B* have been reported previously in canine bladder cancers [14,54]. In the present study *LRP1B* mutations occurred in six samples (17% of the cohort), of which five were UD^V595E^ (18%). *LRP1B*, a putative tumor suppressor gene, encodes a member of the low density lipoprotein (LDL) receptor family, and is among the most frequently altered genes in human cancers, by a variety of both genetic and epigenetic mechanisms [60]. Somatic mutations of *LRP1B* are estimated to occur in 12% of all human cancer cases, and in more than 20% of cases of certain tumor types, including bladder cancers [61]. Furthermore, deletion of the region encoding *LRP1B* has been defined as a hallmark of high grade human UC, with allelic loss of this site in 49% of grade 3 tumors versus 8% of grade 1 tumors [62]. Similarly, *LRP1B* was shown to lie in a region of highly recurrent deletion in canine UC based on prior oaCGH analysis [11], and also in WES-derived read depth analysis in the present study (deleted in 25/28, 89.3%) UD^V595E^ specimens and 8/8 POS^V595E^ specimens, S1 Fig). Of particular note, *LRP1B* mutation has been shown to be a positive predictor of response to immune checkpoint inhibitor therapy in multiple human cancer subtypes [61]. Both *LRP1B* and *RYR2* are therefore logical candidates for consideration as positive predictors of immunotherapy response in canine trials of this treatment modality.

### Mutations within multiple DNA repair genes suggest potential for PARP inhibitor therapy

Three DNA damage response and repair genes were mutated in more than two samples; *MSH6*, *MDC1* and *ATM*. *MDC1* is involved in several processes relating to DNA damage, including checkpoint-mediated cell cycle arrest in response to double-stranded DNA breaks, and activation of ATM Serine/Threonine Kinase. Genomic alterations of *MDC1* in human cancers have been shown to increase sensitivity to DNA damaging chemotherapeutic reagents including doxorubicin and cisplatin [63]. Similarly, ATM acts as a sensor of DNA damage and cell cycle checkpoint via regulation of genes including *TP53* and *BRCA1*. The *ATM* gene is altered in ~6% of all human cancers and in ~11% of human urothelial carcinomas [17], and showed missense mutations in three samples in the present study, all of which were UD^V595E^ specimens. Recent studies in human UC patients [64,65] reported that the presence of *ATM* mutations confers a significantly greater benefit from treatment with immune checkpoint inhibitors, and elevates sensitivity to a total of 29 drug therapies, including the first-line treatment cisplatin. The presence of recurrent mutations of these DNA repair genes in canine UC may therefore open up the possibly for PARP inhibitor therapy in a proportion of cases, as has been suggested for human UC [66].

### Chromatin modifiers and regulators are frequently altered in both canine and human UC

Several studies note a high prevalence of mutations of chromatin modifiers in human UC, including histone demethylases and methyltransferases [18,38,49,53]. Similarly, the present study highlights several epigenetic factors as targets of recurrent mutation in canine UC, particularly histone demethylase and methyltransferase genes and the chromatin remodeling gene *ARID1A*. Mutations in *KDM6A* and *ARID1A* have been reported as the most frequently altered chromatin modifying genes in human UC regardless of tumor stage or grade, suggesting that they are early events [48]. We identified *STAG2* alterations in three samples. *STAG2* is another chromatin regulator that is frequently altered in many human cancers, including UC, in which it acts as a tumor suppressor [67]. One of the primary functions of *STAG2* is in regulating the cohesion and segregation of sister chromatids, and it has been suggested that disruption of this gene may be associated with the high incidence of aneuploidy in human UC. We are not aware of prior reports of *STAG2* alteration in canine UC, but the functional involvement of this gene is plausible given that these tumors show a remarkably high incidence of chromosome imbalance [11,21,68].

### Identification of a conserved *SMCHD1* variant within UD^V595E^ specimens

*SMCHD1* was among the most frequently mutated genes identified in the study. Missense variants were found in 18% of UD^V595E^ samples, but were absent from the eight POS^V595E^ samples. *SMCHD1* plays a role in epigenetic silencing via regulation of chromatin architecture, and in DNA repair in response to double-strand breaks. Mutations of *SMCHD1* are infrequent in human cancers; however, a recent report defined a model in which somatic alterations of three genes, including *SMCHD1*, are predictive of overall survival in human bladder cancer [69]. Interestingly, while human *SMCHD1* shows no evidence of mutational hotspots [38], four canine UC specimens shared the same variant, at the site orthologous to residue N86 in the human gene. This therefore constitutes the second most frequently mutated site in the present study, after *BRAF* V595E.

### Contrasting patterns of somatic mutation in POS^V595E^ and UD^V595E^ canine UC

Although the infrequency of recurrent mutations of the same gene precludes statistical combinatorial analysis, Fig 1 highlights several patterns that warrant further analysis in larger cohorts. At the level of growth factors and ligands that activate the MAPK pathway, mutations of *FGF/FGFR* and *ERBB2* genes were found only in UD^V595E^ samples (5/28, 18%); while *EGF/EGFR* mutations were identified only in POS^V595E^ samples (2/8, 25%). Mutations of the DNA repair genes *ATM* and *MDC1* occurred only in UD^V595E^ samples (six mutations among 5/28 samples, 18%). Among the chromatin remodeling genes *ARID1A*, *SMARCC1*, *PBRM1*, *SMCHD1* and *CHD4/5/6/7* were 19 instances of mutation, of which 17 were found in UD^V595E^ samples. Furthermore, five samples (four of which were UD^V595E^) had mutations in two chromatin remodeling genes. These early findings suggest that UD^V595E^ samples may be enriched in mutations involving DNA repair genes and chromatin-remodeling genes. Of particular note, each of the five samples with *SMCHD1* mutations also showed short in-frame deletions within either *BRAF* or *MAP2K1*. The coincident nature of these events indicates the potential to define additional molecular subtypes within the UD^V595E^ cohort that are based on combinatorial assessment of mutational signatures in multiple genes.

### Short in-frame deletions within *BRAF* and *MAP2K1* are recurrent events in UD^V595E^ canine UC

The most critical finding from the present study is the detection of mutually exclusive short in-frame deletions in the *BRAF* and *MAP2K1* genes in 13/28 (46%) UD^V595E^ samples. Seven samples showed either 9 bp or 15 bp deletions in *BRAF* exon 12. This region encodes the β3-αC loop of the kinase domain, which human studies show to be involved in the mechanism that allows BRAF to switch between an active and an inactive state. Deletions that induce shortening of the loop limit the conformational flexibility of the protein, locking it in a RAS-independent, constitutively active form, with kinase activity reaching a peak with the deletion of five amino acids from the β3-αC loop [70]. These mutations may therefore indicate alternative mechanisms for MAPK pathway activation in canine UC, aside from the *BRAF* V595E point mutation.

While rare in general, in-frame deletions within *BRAF* exon 12 have been reported in a small number of human cancer subtypes, the majority of which eliminate the region extending from amino acid residue N486 to P490 (termed ∆NVTAP, Fig 2). This human deletion variant (h∆NVTAP, assigned COSMIC Genomic Mutation ID COSV56100024) is equivalent to elimination of N474-P478 from canine BRAF (c∆NVTAP), which was the most common of these deletions identified in the present study. Similarly, the 15 bp deletion identified in UD-112 (c∆LNVTAP > F) is orthologous to the previously described human variant with COSMIC Genomic Mutation ID COSV104608678 (h∆LNVTAP > F). One study [70] reported *BRAF* in-frame deletions in ~1% of human pancreatic carcinomas, of which 11 were of the ∆NVTAP type. A recent study identified ∆NVTAP in 20/69 (29%) cases of human adult Langerhans cell histiocytosis (LCH), an inflammatory myeloid neoplasm that is strongly linked to aberrant RAF-MEK-ERK activation [71]. Interestingly, 25/69 (36%) cases from the same human LCH cohort harbored the *BRAF* V600E mutation, but only a single case presented with both of these variants concurrently. Sporadic examples of *BRAF* exon 12 in-frame deletions have also been reported in human myeloid neoplasms, lung carcinomas, colon carcinomas and prostatic carcinomas, in which they are mutually exclusive from V600E mutations, and also from mutations of RAS genes [70,72–79].

In human cancers, the ∆NVTAP variant exhibits MAPK pathway signaling activity comparable to that of *BRAF* V600E [70]. Functional studies using human cancer cell lines have shown that, in contrast to V600E mutants, ∆NVTAP mutants are resistant to BRAF monomer inhibitors such as vemurafenib and dabrafenib that target the ‘αC-out/DFG-in’ structural conformation of the active protein kinase. Instead, mutants with exon 12 in-frame deletions are susceptible to inhibitors that target the ‘αC-in/DFG-out’ conformation, such as the pan-RAF dimer inhibitors AZ628 and LY3009120, as well as allosteric MEK inhibitors such as cobimetinib and trametinib [70,73,74,80]. Moreover, introduction of the ∆NVTAP alteration into human cells bearing the *BRAF* V600E mutation has been shown to confer resistance to vemurafenib *in vitro*, reinforcing the significance of this deletion for therapeutic response [70]. Consequently, there is increasing emphasis on classification of *BRAF* alterations in human tumors into categories that are predictive of response to different therapeutic compounds, based on the specific protein conformations they target [35].

We examined other genes within the MAPK pathway for evidence of similar alterations, and identified two intervals exhibiting short in-frame deletions in the dog *MAP2K1* gene. *MAP2K1* encodes the MEK1 protein kinase, which is activated by BRAF and which subsequently activates the downstream ERK protein, stimulating cell growth, proliferation and survival. MEK therefore offers an alternative mechanism for inducing aberrant ERK activation in the absence of mutant BRAF. Four mutational hotspots have been identified in human MEK1. Two of these, spanning exons 2 and 3, harbor short deletions in a small subset of cancers, namely the negative regulatory region known as inhibitory helix A, and the catalytic β3-αC loop region of the kinase domain [81]. While generally infrequent, oncogenic deletions within *MAP2K1* exons 2 and 3 are enriched in a small subset of human cancers including certain melanocytic lesions and particularly pediatric LCH (~30% of cases), and are mutually exclusive from the *BRAF* V600E variant [73,82–86]. These two hotspots of *MAP2K1* deletion in human cancers are orthologous to the regions of recurrent deletion identified in our canine UC cohort.

Normally, the negative regulatory region interacts with the kinase domain of the MEK1 protein, causing it to become stabilized in an inactive conformation; thus disruption of the negative regulatory region can trigger MEK1 kinase activity. Functional analyses have shown that a variety of point mutations and deletions within this region result in aberrant ERK phosphorylation consistent with constitutive activity. This includes the K57E variant that we identified in a single canine sample, which corresponds to the most frequently altered *MAP2K1* codon identified in human cancers, and which has been associated with emergence of resistance to BRAF monomer inhibitors in human cancer cells [38]. Similarly, the canine ∆FLTQKQ > L and ∆QKQKVG variants identified in the present study are orthologous to human ∆FLTQKQ > L (spanning MEK1 residues F53-Q58) and ∆QKQKVG (Q57-G61), which have been assigned COSMIC Genomic Mutation IDs COSV61072289 and COSV61072263, respectively [87]. Deletions spanning the negative regulatory region have been associated with the onset of resistance to BRAF monomer inhibitors in human patients, but tumors with these variants are typically responsive to allosteric MEK1 inhibitors and ERK inhibitors [88].

The mutational hotspot within human *MAP2K1* exon 3 is highly conserved with the β3-αC loop region of BRAF, as well as other protein kinases including EGFR and HER2 [70,74]. Similarly, deletion within these regions cause shortening of the loop to yield an activated conformation, leading to RAF-independent MAPK pathway activation at a level determined both by the size and the site of the deletion [81]. Deletions within the MEK1 catalytic kinase domain confer variable response to different MEK inhibitors, and can lead to resistance to allosteric MEK1 inhibitor therapy. Promising results have been observed using ATP-competitive selective MEK inhibitors and ERK inhibitors to target these mutations, as well as those within the negative regulatory region [88]. Thus, somatic deletions within the β3-αC loop regions of RAF and MEK proteins confer a closely related impact on drug susceptibility in human cancers. In turn, as with *BRAF*, *MAP2K1* mutations are increasingly categorized according to their impact on MEK1 conformation and consequent therapeutic response.

**Fig 4:**
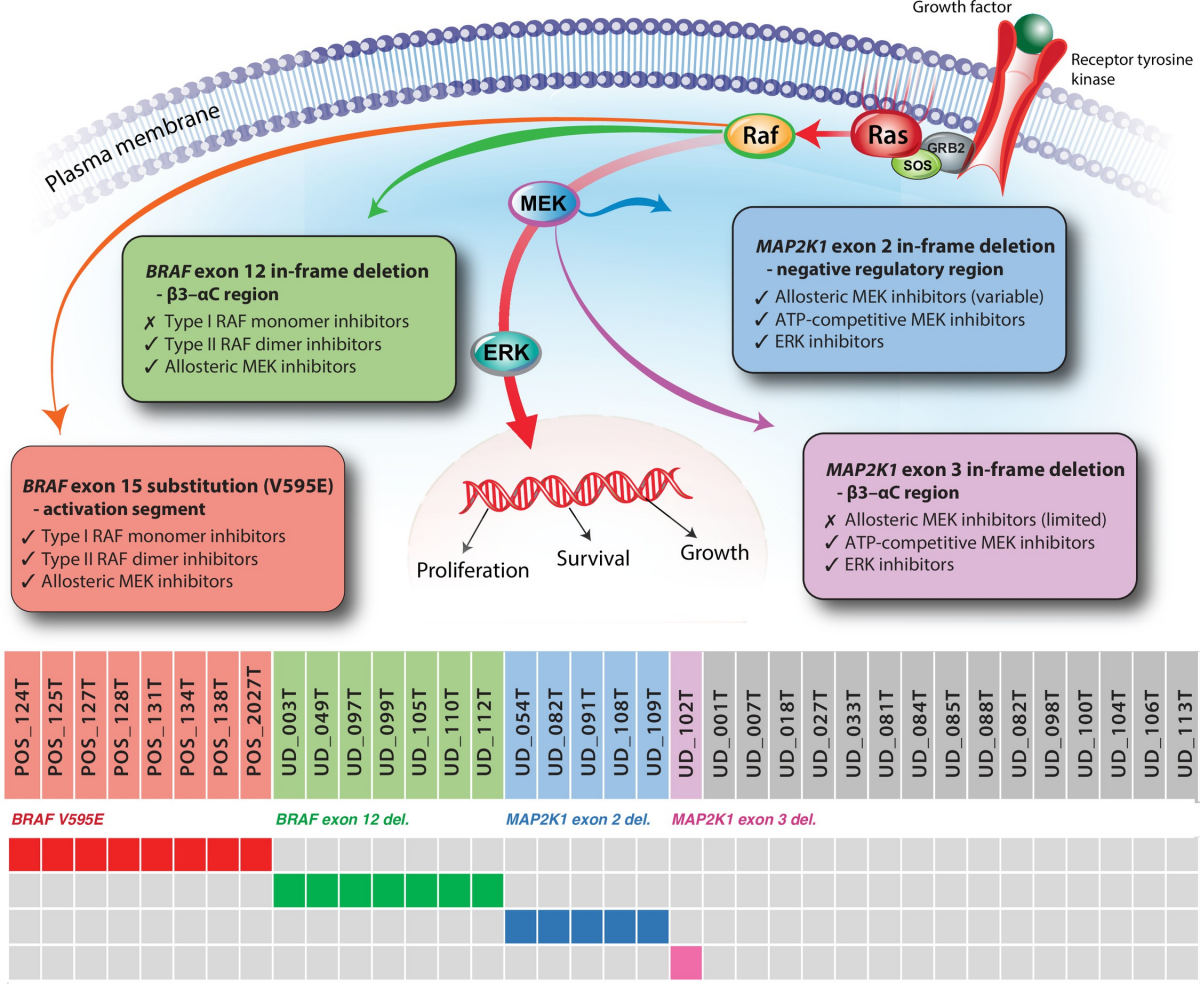
Potential opportunities for using molecular subclassification as a mechanism for treatment stratification. This simplified oncoplot shows the distribution of *BRAF* and *MAP2K1* alterations within the sample cohort, shown in context with the site of action of RAF and MEK in the MAPK pathway. The four categories of variants are annotated to show potential therapeutic strategies, based on extrapolation of data from human studies.

These findings identify potential mechanisms for MAPK pathway activation in canine UC without *BRAF* V595E mutation that may also have therapeutic implications (Fig 4). The absence of detectable mutations in MAPK pathway members within the remaining cases may be for a variety of reasons. Mutations may exist with a VAF below the limit of detection of WES analysis, or may occur in regions that are not captured effectively by the exome baits used in this study. Pathway disruption may be induced by mechanisms other than somatic gene mutation, such as the altered activity of distant regulatory elements or methylation. Alternatively, these may represent molecularly distinct forms of canine UC arising through dysregulation of different pathways.

### Study advantages and limitations

Aside from *BRAF* V595E there is relatively limited evidence for recurrent alterations shared between published studies of canine UC. It is likely that a combination of factors explains this limited overlap, including the use of different sample types (fresh vs. fixed, archival tissue vs. urine sediment), experimental strategies (WES vs targeted amplicon sequencing vs RNAseq) and methodologies for variant detection and filtering. Additional shared variants may become evident as studies transition toward the use of new dog genome assemblies, which are derived from different individuals and which provide more comprehensive sequence annotation of non-protein-coding regions [89]. This study adds the additional factor of an intentional skew toward specimens without the *BRAF* V595E mutation. The use of urine-derived DNA samples for WES analysis allowed us avoid formalin-induced sequence artefacts that can be misclassified as somatic mutations. Specimens derived from free-catch urine do not, however, allow determination of the primary site of origin of the tumor, and so it is possible that our study cohort includes lesions from different sites within the urogenital tract. Given the propensity for late-stage diagnosis with local invasion and distant metastasis, however, this confounding factor extends also to histopathologically-validated biopsies, particularly those retrieved at necropsy. In an earlier ddPCR study of 60 abnormal biopsies from the canine urogenital tract with a diagnosis other than UC, eight (13%) showed a relative copy number increase of cfa13 and only a single sample (2%) showed gain of cfa36 [90]. None of the 60 samples showed gain of both chromosomes, providing additional support for the presence of a urothelial carcinoma in free-catch urine samples that test positive for both CNV signatures. Additional support for the presence of UC was provided by the derivation of DNA copy number profiles from WES read depth data (S1 Fig). This indicated that the genomic imbalances evident in specimens from the present study strongly recapitulate those detected in our microarray-based investigations of an independent cohort of canine UC cases [68]. This provides the opportunity to use WES data for detecting clinically predictive CNA signatures, which may reflect drivers of disease pathogenesis, therapeutic targets and/or markers of distinct UC subtypes.

The method and criteria by which cases have been classified as lacking the *BRAF* V595E mutation varies between studies, and have included conventional Sanger sequencing analysis, detection of restriction fragment length polymorphisms, targeted amplicon resequencing, and WES analysis [4]. Sanger sequencing analysis is generally considered to have a sensitivity limit of ~15%, while next-generation sequencing methods (where sensitivity is heavily dictated by the read-depth and filtering criteria applied) frequently sets a threshold of 5% VAF. Our study benefits from classification by ddPCR analysis using a methodology we have shown to detect mutations reliably down to a fractional abundance of 0.025%. Since the confidence with which absence of a variant can be reported is heavily dependent on the nature and analysis parameters of the detection method used, we elect to use the term *‘undetected’* instead of ‘*wild-type*’.

Integration of data from prior studies is confounded by inconsistencies in the definition of tumor types as ‘bladder cancer’, ‘transitional cell carcinoma’ and ‘urothelial carcinoma’, with these terms sometimes used interchangeably in the same report. Furthermore, canine bladder cancers have a propensity for invading into adjacent tissues such as the ureter, the prostate gland and the prostatic urethra. Coupled with the fact that these lesions are typically encountered at a late stage, it is challenging to identify the true primary site of origin of the malignancy within the urinary tract, even with full necropsy evaluation. In males, this is complicated further by the potential for a prostatic origin, and indeed it has been estimated that 30% of invasive UC cases in male dogs have prostatic involvement [1,2]. To date there are few genomic studies focusing specifically on canine prostate cancers and so additional work is needed to catalog their molecular profiles and to determine whether they exhibit molecular signatures distinct from those of bladder tumors.

## Conclusions

Our study suggests that the ~15% of canine UC cases without *BRAF* V595E mutation do not harbor a variant of comparable prevalence elsewhere within this gene, nor in the coding region of any other gene within the exome captured. To date little is known of the clinical, anatomical, histologic, and prognostic significance of canine UC in which the *BRAF* V595E variant is undetected. It remains to be determined whether the absence of this mutation in a minority of cases indicates earlier-stage disease that will eventually develop the variant, or whether it constitutes one or more distinct molecular subtypes with somatic alterations in other genes activating alternative pathways. Armed with additional markers for subclassification of canine UC we can now begin to correlate discrete molecular signatures with clinical and histologic features that impact tumor behavior and therapeutic response. This also provides a mechanism to track the accumulation of key somatic alterations from tumor initiation through to progression, to map out the relative order and timeline during which these events emerge. We noted that the breeds represented in the cohort of UD^V595E^ cases showed only modest overlap with those considered to be at high risk of UC (9/28 dogs, comprising seven beagles, one Scottish terrier and one beagle mix). The availability of new markers for UD^V595E^ cases will allow us to investigate, in a larger sample size, whether UD^V595E^ UC and POS^V595E^ UC represent distinct molecular subtypes with differential breed predisposition.

Our findings raise the possibility that short in-frame deletions within *BRAF* exon 12 and *MAP2K1* exons 2 and 3 are MAPK-pathway activating events that may have significant therapeutic implications for canine UC. To this end, the combinatorial CE assay described in the present study provides a rapid, cost-effective and non-invasive screening strategy with potential as a companion diagnostic for veterinary medicine. When considered in context with clinical signs, this may also have utility as a means to monitor dogs during treatment for emergence of these alterations, signaling loss of chemotherapeutic sensitivity and prompting the need to pursue alternative treatments. Logistical and financial challenges may preclude the routine use of alternative MAPK pathway inhibitor therapies in a veterinary setting; however, the identification of canine *BRAF* and *MAP2K1* in-frame deletions may expedite the association of mutational status with drug response, given the relative infrequency of these variants in human tumors. The reported prevalence of *BRAF* and *MAP2K1* in-frame deletions in human cancers is thought likely to be an underestimate since sequencing technologies and analytic tools are designed primarily for identification of point mutations [74]. In-frame deletions comprise only 4.3% of all *MAP2K1* mutations reported across all human cancers, and only 0.2% of all *BRAF* mutations [38]; consequently, they remain relatively understudied. The conservation of *BRAF* and *MAP2K1* deletions in canine UC and human LCH and pancreatic carcinomas suggests there may be synergistic benefit from parallel functional studies of these diverse cancer types, as a means to better understand the broader relationship between somatic alteration, protein conformation and therapeutic sensitivity.

## Supporting information

S1 Fig Generation of DNA copy number profiles from WES read depth data. Chromosome location is indicated on the horizontal axis. A) Comparison of DNA copy number profiles for the same sample derived from i) oligonucleotide-array comparative genomic hybridization (oaCGH) analysis and ii) WES read depth data. Chromosome location is indicated on the horizontal axis, and the vertical axis shows pseudo-log ratios of read depth from the sample versus the non-neoplastic reference pool. Blue and red horizontal lines immediately above and below the midline indicate thresholds for classification of relative DNA copy number gains and losses, respectively. Data for each chromosome are shown in a different color to aid interpretation. This example demonstrates that WES-derived data generate profiles that strongly recapitulate those derived by oaCGH analysis. The characteristic UC signature of gain of cfa13 and 36, and loss of cfa19, is evident in both profiles. A strongly conserved, complex pattern of high-amplitude copy number gains is also evident on cfa10, interspersed with regions of balanced copy number and deletion. Cfa7 and 12 also show copy number complexity suggestive of structural rearrangement. These observations support the use of WES read depth data for the assessment of genomic instability. B) DNA copy number profiles derived from WES read depth data for POS^V595E^ and UD^V595E^ specimens (this study), compared to data from an independent cohort profiled using oaCGH analysis [11]. Penetrance plots for each sample type and analysis method indicate the percentage of samples within that subgroup that shared the same CNA (vertical axis). Genomic gains are shown in blue and losses in red. These observations support the presence of exfoliated urothelial carcinoma cells in the urine of the dogs included in this study.
(PDF)

S2 Fig Summary of variants identified in each sample. A) The total number of variants identified in each sample is shown as a stacked column plot, with each column subdivided to indicate the number of variants of each category. B) The same information is shown as a 100% stacked column plot, with each column subdivided to show the percentage contribution of variants of each category as a proportion of all variants identified. POS^V595E^ samples (n = 8) are denoted by aqua shading and UD^V595E^ samples (n = 28) by purple shading. The sex of each dog is indicated by a colored box above the sample code (females shown in pink, males in blue, and dogs of unknown sex shown in grey). Within that box, neuter status is shown as either N (neutered male), S (spayed female) or I (intact).
(PDF)

S3 Fig Statistical comparison of the number of mutations identified in POS^V595E^ vs UD^V595E^ samples. a) Comparison of the total number of mutations identified in POS^V595E^ vs UD^V595E^ samples. The grey horizontal line shows the mean number of mutations identified across all 36 samples combined, and blue dotted lines indicate the mean value within each sample group. Quantile box plots (shown in red) summarize the variation in the number of mutations identified within each of the two sample groups. The chart is annotated to show the p-values obtained for the comparison of the mean number of mutations identified in each sample group (two-sample t test), and for the variance in the number of mutations identified in each sample group (two-sided F test). b)-e) show similar charts for comparisons of the number of mutations of each category observed in each sample group.
(PDF)

S4 Fig Comparison of *BRAF* V595E variant frequency obtained using different techniques. The X axis indicates the fractional abundance of the mutant allele in nine specimens as determined by ddPCR analysis, and the Y axis shows the variant allele frequency of the mutation in the same samples as determined by whole exome sequencing analysis. The dashed line indicates the line of best fit, demonstrating the strong correlation in the data obtained by both methods (R^2^ value = 0.979) across a wide range of values. The sample denoted as control has a 4.3% fractional abundance of the *BRAF* V595E variant as determined by ddPCR, and was included in whole exome analysis solely for comparison of mutant allele frequencies generated by both methods at the low end of the range of values.
(PDF)

S5 Fig Validation of in-frame deletions in *BRAF* exon 12. Aligned Sanger sequencing traces for the seven UD^V595E^ samples support the short deletions identified in *BRAF* exon 12 using WES analysis. Each sample was sequenced from both directions flanking the aberrant interval, which is highlighted in yellow. The resulting amino acid change is shown to the right. A trace from a non-neoplastic control sample is shown at the top of the alignment for comparison.
(PDF)

S1 Table Primer sequences and predicted amplicon sizes. *BRAF* and *MAP2K1* target regions are listed with the corresponding primer sequences and 5’ fluorophore tag. The underlined sequence represents the 17 nucleotides that allow size discrimination between the *BRAF* V595 wild-type and mutant alleles. The lower case nucleotide represents the additional sequence mismatch introduced into the forward primer for the mutant allele to prevent spurious binding with the wild-type allele. The last two columns indicate the expected amplicon size obtained from wild-type alleles, and the observed amplicon sizes from the study cohort.
(XLSX)
