# Supplementary figures and images for: Whole exome sequencing analysis of canine urothelial carcinomas without BRAF V595E mutation: Short in-frame deletions in BRAF and MAP2K1 suggest alternative mechanisms for MAPK pathway disruption
Source: PLoS Genet. 2023 Apr 20;19(4):e1010575. doi: 10.1371/journal.pgen.1010575 (PMC10153751; doi:10.1371/journal.pgen.1010575)

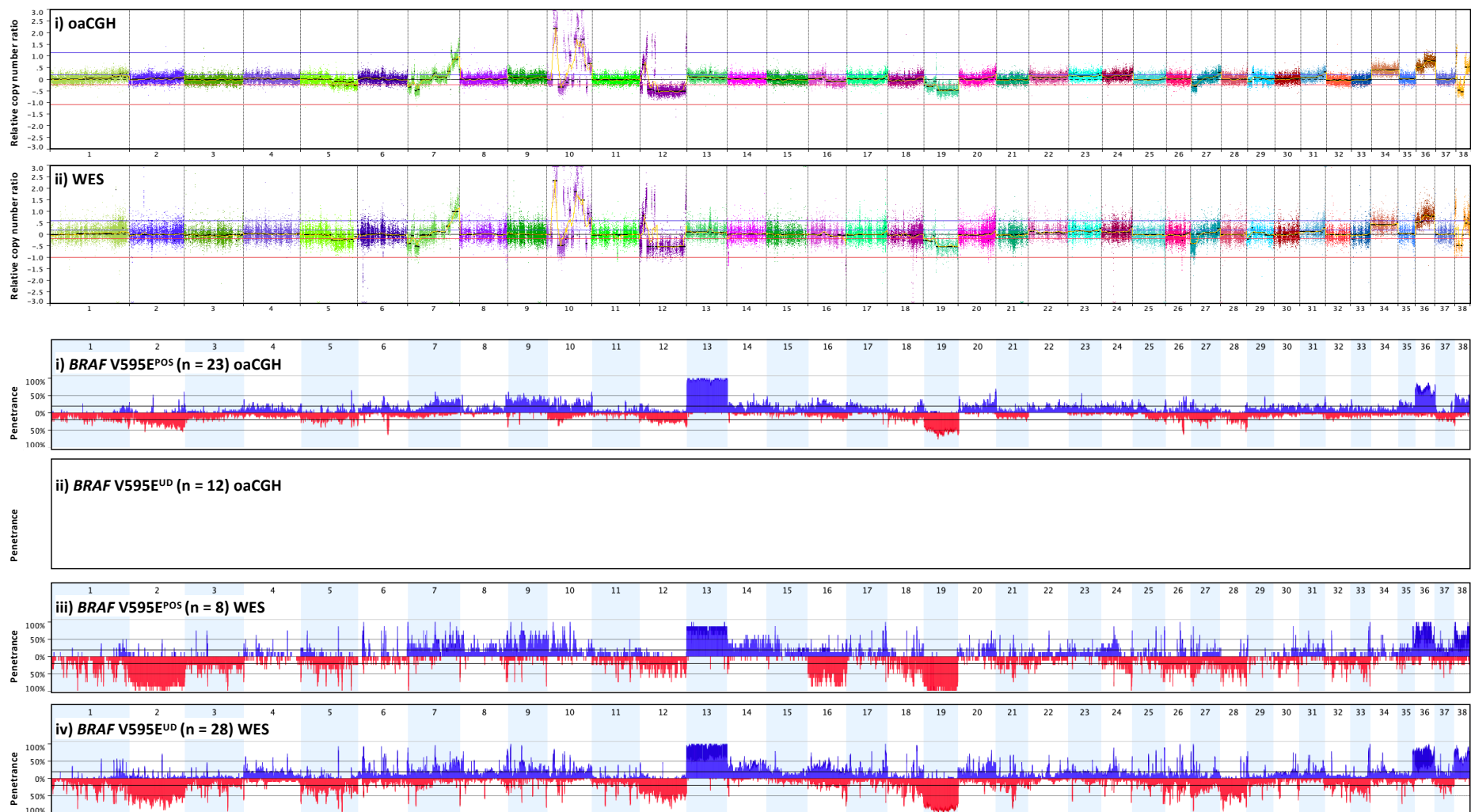

Figure S1

Supplement: S1 Fig — Chromosome location is indicated on the horizontal axis. A) Comparison of DNA copy number profiles for the same sample derived from i) oligonucleotide-array comparative genomic hybridization (oaCGH) analysis and ii) WES read depth data. Chromosome location is indicated on the horizontal axis, and the vertical axis shows pseudo-log ratios of read depth from the sample versus the non-neoplastic reference pool. Blue and red horizontal lines immediately above and below the midline indicate thresholds for classification of relative DNA copy number gains and losses, respectively. Data for each chromosome are shown in a different color to aid interpretation. This example demonstrates that WES-derived data generate profiles that strongly recapitulate those derived by oaCGH analysis. The characteristic UC signature of gain of cfa13 and 36, and loss of cfa19, is evident in both profiles. A strongly conserved, complex pattern of high-amplitude copy number gains is also evident on cfa10, interspersed with regions of balanced copy number and deletion. Cfa7 and 12 also show copy number complexity suggestive of structural rearrangement. These observations support the use of WES read depth data for the assessment of genomic instability. B) DNA copy number profiles derived from WES read depth data for POSV595E and UDV595E specimens (this study), compared to data from an independent cohort profiled using oaCGH analysis [11]. Penetrance plots for each sample type and analysis method indicate the percentage of samples within that subgroup that shared the same CNA (vertical axis). Genomic gains are shown in blue and losses in red. These observations support the presence of exfoliated urothelial carcinoma cells in the urine of the dogs included in this study. (PDF) [file pgen.1010575.s001.pdf]

A

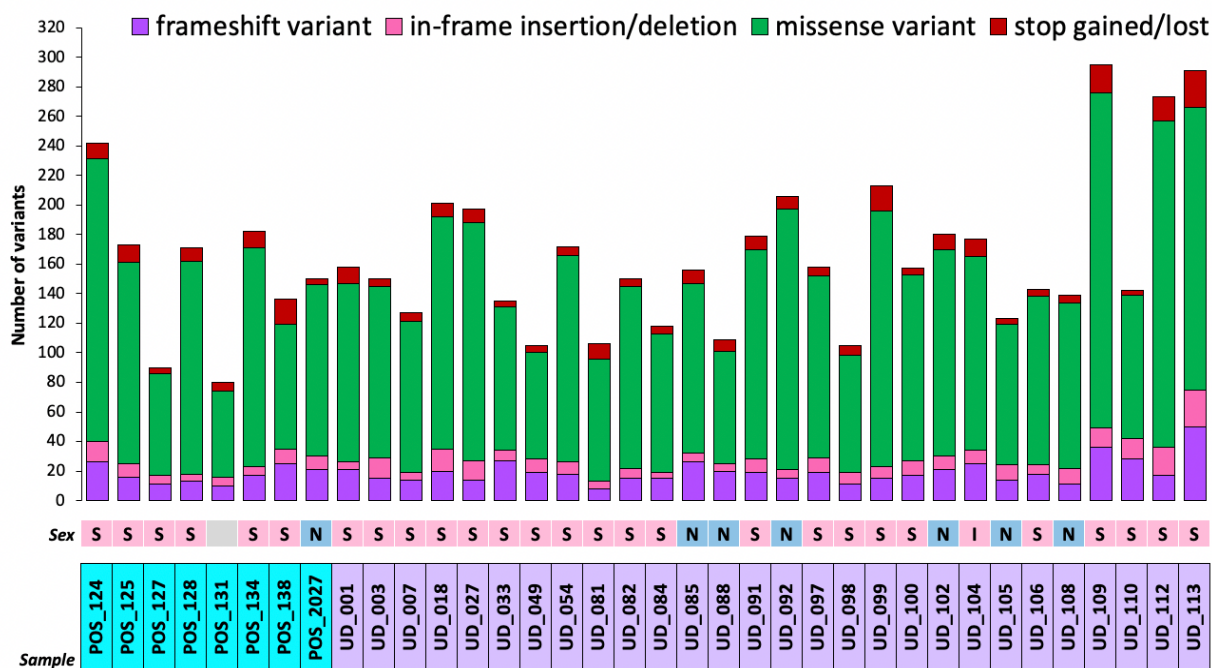

B

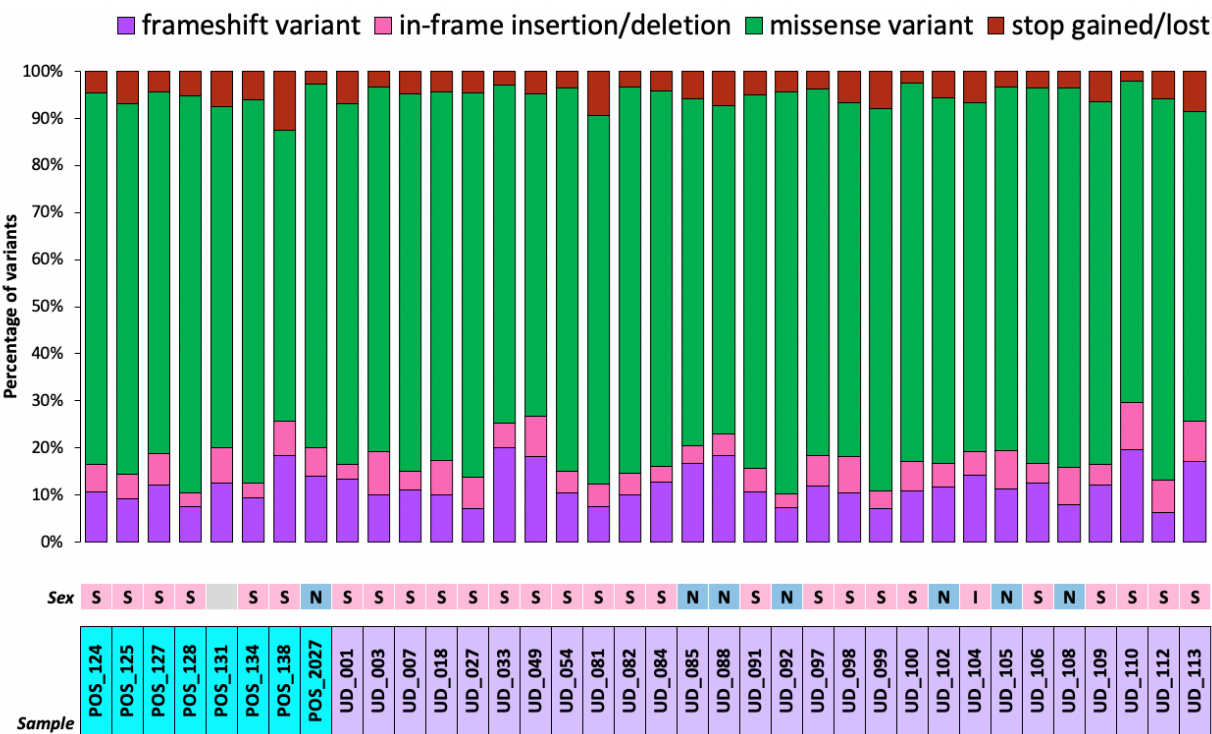

Figure S2

Supplement: S2 Fig — A) The total number of variants identified in each sample is shown as a stacked column plot, with each column subdivided to indicate the number of variants of each category. B) The same information is shown as a 100% stacked column plot, with each column subdivided to show the percentage contribution of variants of each category as a proportion of all variants identified. POSV595E samples (n = 8) are denoted by aqua shading and UDV595E samples (n = 28) by purple shading. The sex of each dog is indicated by a colored box above the sample code (females shown in pink, males in blue, and dogs of unknown sex shown in grey). Within that box, neuter status is shown as either N (neutered male), S (spayed female) or I (intact). (PDF) [file pgen.1010575.s002.pdf]

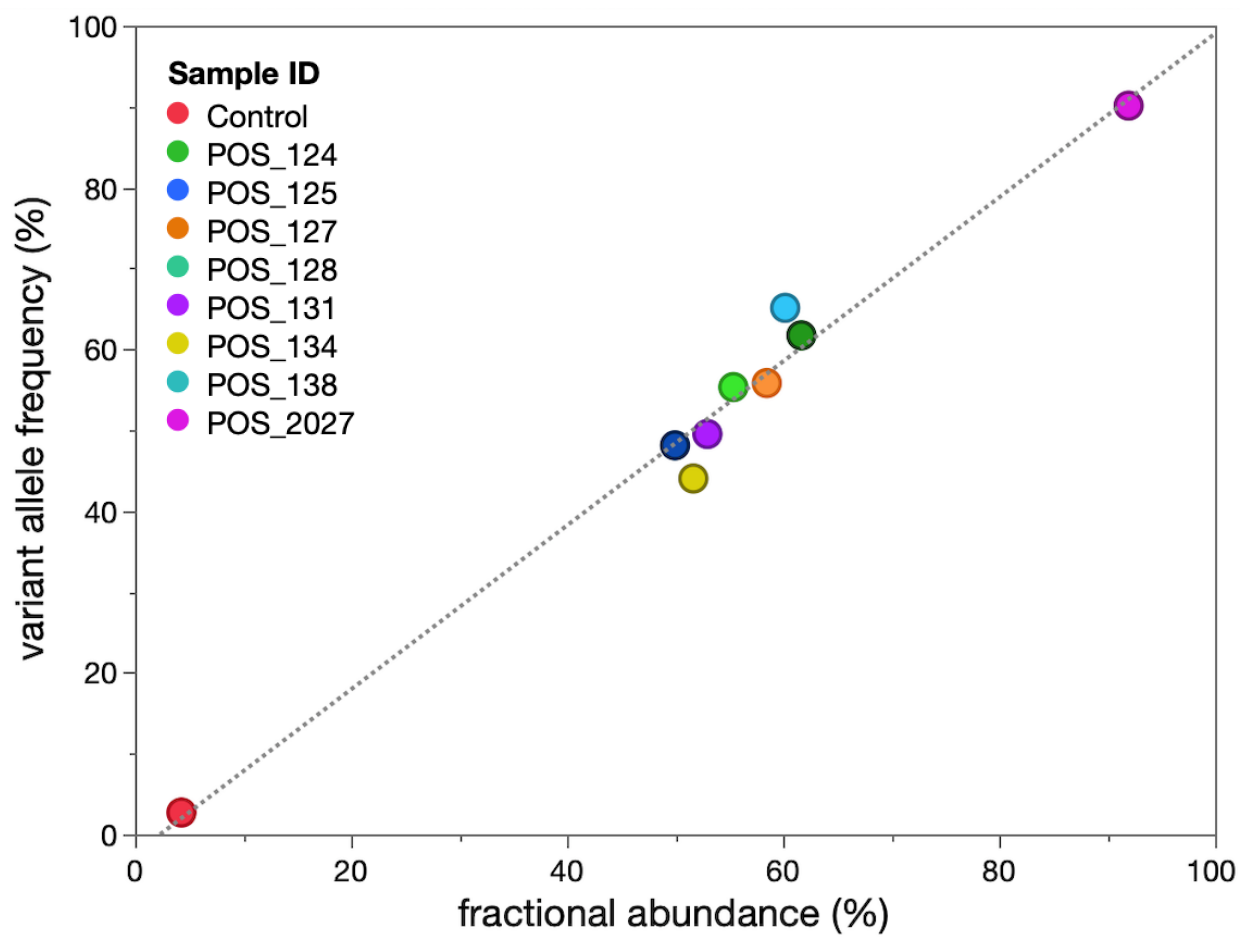

Figure S4

Supplement: S4 Fig — The X axis indicates the fractional abundance of the mutant allele in nine specimens as determined by ddPCR analysis, and the Y axis shows the variant allele frequency of the mutation in the same samples as determined by whole exome sequencing analysis. The dashed line indicates the line of best fit, demonstrating the strong correlation in the data obtained by both methods (R2 value = 0.979) across a wide range of values. The sample denoted as control has a 4.3% fractional abundance of the BRAF V595E variant as determined by ddPCR, and was included in whole exome analysis solely for comparison of mutant allele frequencies generated by both methods at the low end of the range of values. (PDF) [file pgen.1010575.s004.pdf]

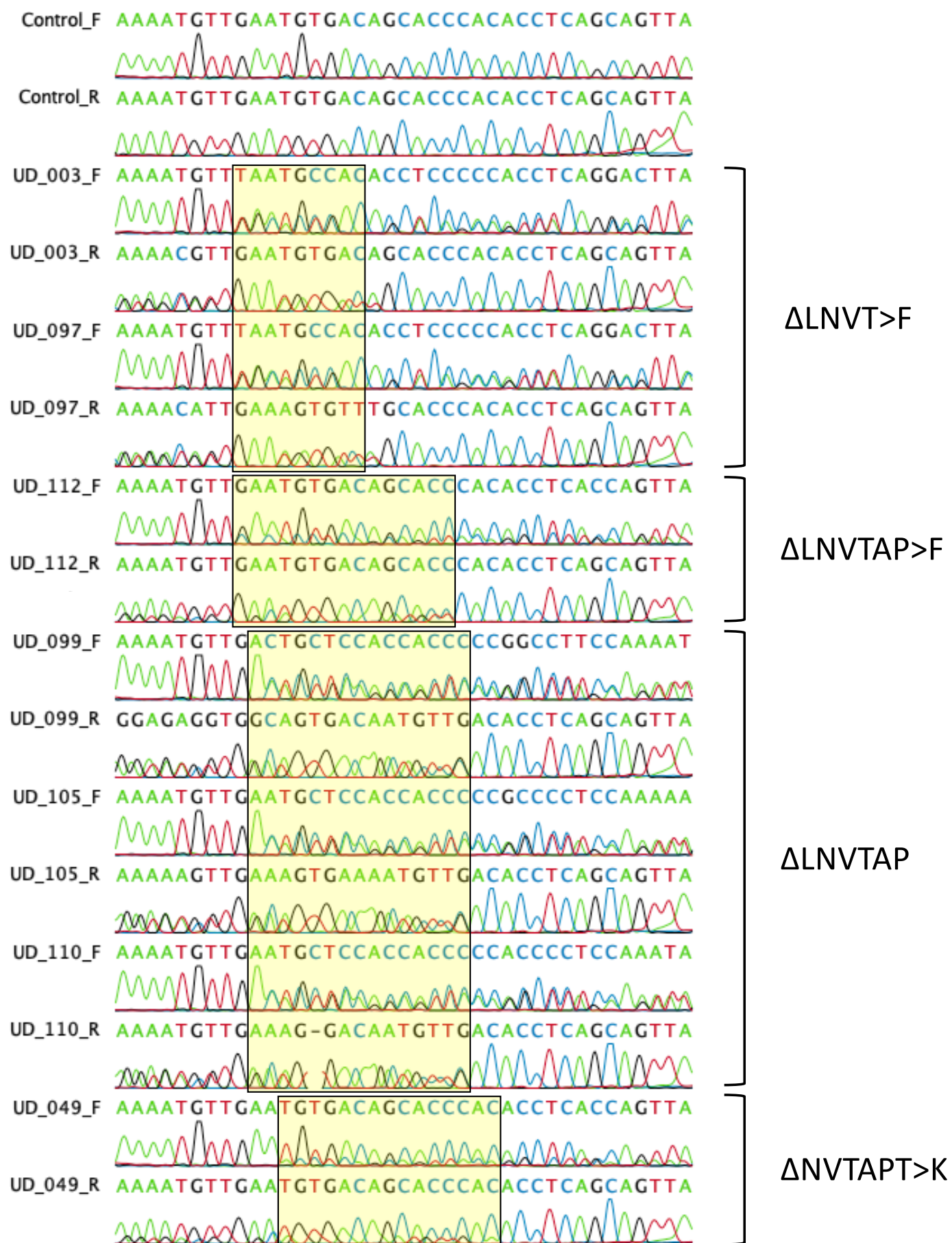

Figure S5

Supplement: S5 Fig — Aligned Sanger sequencing traces for the seven UDV595E samples support the short deletions identified in BRAF exon 12 using WES analysis. Each sample was sequenced from both directions flanking the aberrant interval, which is highlighted in yellow. The resulting amino acid change is shown to the right. A trace from a non-neoplastic control sample is shown at the top of the alignment for comparison. (PDF) [file pgen.1010575.s005.pdf]
