# Supplementary material for: Whole exome sequencing analysis of canine urothelial carcinomas without BRAF V595E mutation: Short in-frame deletions in BRAF and MAP2K1 suggest alternative mechanisms for MAPK pathway disruption
Source: PLoS Genet. 2023 Apr 20;19(4):e1010575. doi: 10.1371/journal.pgen.1010575 (PMC10153751; doi:10.1371/journal.pgen.1010575)
Supplement: S3 Fig — a) Comparison of the total number of mutations identified in POSV595E vs UDV595E samples. The grey horizontal line shows the mean number of mutations identified across all 36 samples combined, and blue dotted lines indicate the mean value within each sample group. Quantile box plots (shown in red) summarize the variation in the number of mutations identified within each of the two sample groups. The chart is annotated to show the p-values obtained for the comparison of the mean number of mutations identified in each sample group (two-sample t test), and for the variance in the number of mutations identified in each sample group (two-sided F test). b)-e) show similar charts for comparisons of the number of mutations of each category observed in each sample group. (PDF) [file pgen.1010575.s003.pdf]

a) Total number of variants

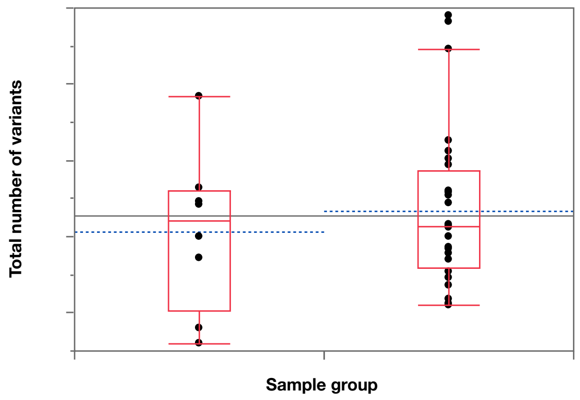

Comparison of means (two-sample t test, p = 0.52)
Comparison of variance (two-sided F test, p = 1.00)

b) Missense variants

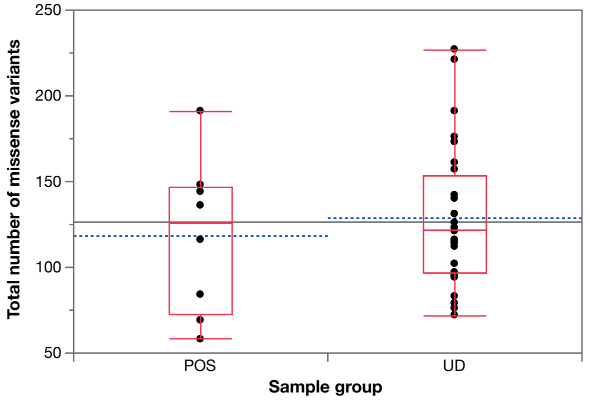

Comparison of means (two-sample t test, p = 0.54)
Comparison of variance (two-sided F test, p = 0.64)

c) In-frame insertions/deletions

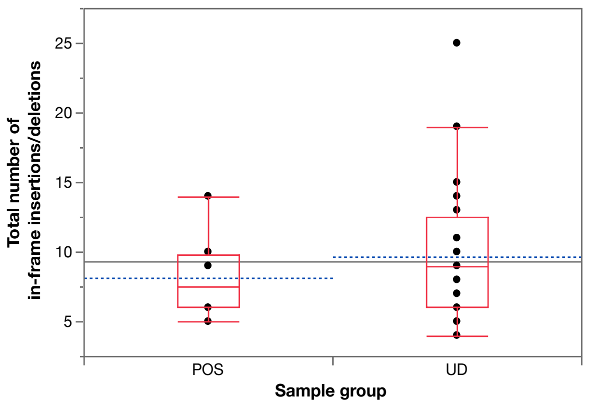

Comparison of means (two-sample t test, p = 0.42)
Comparison of variance (two-sided F test, p = 0.22)

d) Frameshift variants

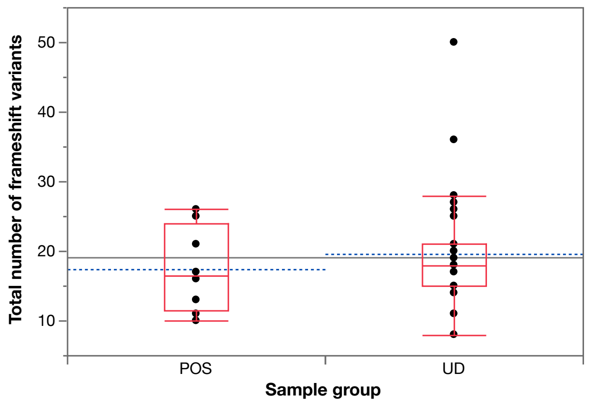

Comparison of means (two-sample t test, p = 0.50)
Comparison of variance (two-sided F test, p = 0.40)

e) Stop gained/lost variants

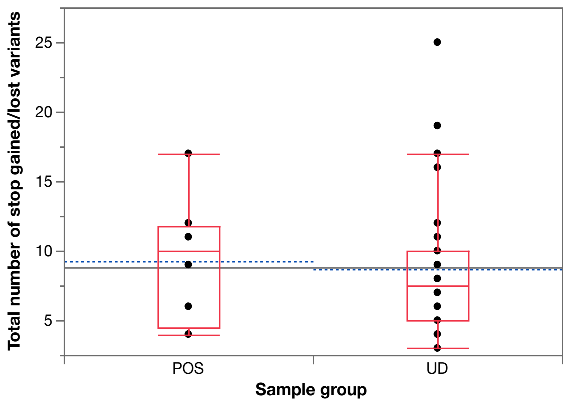

Comparison of means (two-sample t test, p = 0.78)
Comparison of variance (two-sided F test, p = 0.73)

Figure S3
